# Conserved 3′ UTR stem-loop structure in L1 and Alu transposons in human genome: possible role in retrotransposition

**DOI:** 10.1186/s12864-016-3344-4

**Published:** 2016-12-03

**Authors:** Daria Grechishnikova, Maria Poptsova

**Affiliations:** 1 Physics Department, Lomonosov Moscow State University, Moscow, 119991 Russia; 2 National Research University Higher School of Economics, Moscow, 101000 Russia

**Keywords:** L1, Alu, LINE, SINE, Mechanisms of retrotransposition, Stem-loop, Stem-loop structures

## Abstract

**Background:**

In the process of retrotransposition LINEs use their own machinery for copying and inserting themselves into new genomic locations, while SINEs are parasitic and require the machinery of LINEs. The exact mechanism of how a LINE-encoded reverse transcriptase (RT) recognizes its own and SINE RNA remains unclear. However it was shown for the stringent-type LINEs that recognition of a stem-loop at the 3′UTR by RT is essential for retrotransposition. For the relaxed-type LINEs it is believed that the poly-A tail is a common recognition element between LINE and SINE RNA. However polyadenylation is a property of any messenger RNA, and how the LINE RT recognizes transposon and non-transposon RNAs remains an open question. It is likely that RNA secondary structures play an important role in RNA recognition by LINE encoded proteins.

**Results:**

Here we selected a set of L1 and Alu elements from the human genome and investigated their sequences for the presence of position-specific stem-loop structures. We found highly conserved stem-loop positions at the 3′UTR. Comparative structural analyses of a human L1 3′UTR stem-loop showed a similarity to 3′UTR stem-loops of the stringent-type LINEs, which were experimentally shown to be recognized by LINE RT. The consensus stem-loop structure consists of 5–7 bp loop, 8–10 bp stem with a bulge at a distance of 4–6 bp from the loop. The results show that a stem loop with a bulge exists at the 3′-end of Alu. We also found conserved stem-loop positions at 5′UTR and at the end of ORF2 and discuss their possible role.

**Conclusions:**

Here we presented an evidence for the presence of a highly conserved 3′UTR stem-loop structure in L1 and Alu retrotransposons in the human genome. Both stem-loops show structural similarity to the stem-loops of the stringent-type LINEs experimentally confirmed as essential for retrotransposition. Here we hypothesize that both L1 and Alu RNA are recognized by L1 RT via the 3′-end RNA stem-loop structure. Other conserved stem-loop positions in L1 suggest their possible functions in protein-RNA interactions but to date no experimental evidence has been reported.

**Electronic supplementary material:**

The online version of this article (doi:10.1186/s12864-016-3344-4) contains supplementary material, which is available to authorized users.

## Background

L1 family is by its mass the largest family of Long INterspersed Elements (LINEs) in humans; L1s are present in more than 500 000 copies and occupy approximately 17% of the total human genome length [1]. Most of L1s found in the human genome are 5′-end truncated or damaged and therefore have lost the retrotransposition ability [2]. It is estimated that, on average, there are 80–100 L1s in a human being, which are still able to move through the genome [3]. A typical L1 is about 6 kb in length and consists of a 5′ untranslated region (UTR), two non-overlapping open reading frames (ORFs), a 3′ UTR and a poly-A tail.

L1 transcription is initiated by an RNA polymerase II promoter located at the 5′ UTR [4, 5]. The first open reading frame (ORF1) encodes a 40 kDa protein (ORF1p) consisting of a coiled-coil domain [6], a non-canonical RNA recognition motif domain [7, 8] and a basic carboxyl-terminal domain [9]. Although the exact role of ORF1 is not clear, it was demonstrated that this protein is required for retrotransposition [10]. The second protein, ORF2, is a 150 kDa protein and it combines endonuclease (EN) [11] and reverse transcriptase (RT) [12] activities. The L1 3′UTR is about 200 bp and is poorly conserved. It contains a polypurine tract that potentially could form a G-quadruplex structure [13], but the function of this sequence remains unknown. L1 ends with a poly-A tail that was shown to be critical for L1 retrotransposition [14]. Short direct repeats, flanking a transposon, are generated from the target DNA sequence during the L1 integration. The length of repeats can range from a few to several hundred nucleotides [2].

The second family of retrotransposons by mass in the human genome is Alu, belonging to the class of Short Interspersed Elements (SINEs). By copy number Alu is the most ubiquitous retrotransposon with more than 1 mln copies present in the human genome and occupying approximately 11% of the total genome length [1]. The length of a typical Alu is around 300 bp. Though almost all SINEs are derived from tRNA [15], Alus are derived from 7SL RNA, which functions as a component of the signal recognition particle [16, 17]. It consists of two monomers, each similar to the 7SL RNA, an A-rich connecting domain and a poly-A tail varying in length. In contrast to L1 Alu cannot amplify by itself as it does not encode any proteins and hires the L1 retrotransposition machinery [18, 19].

The mechanism of how L1 RT recognizes L1 and Alu RNA remains an open question. Since SINEs borrow retrotransposition enzymatic machinery from LINEs, the more general question is how RT recognizes LINE and SINE RNAs. The similarity between LINE and SINE elements was first shown for the turtle genome: it was found that elements from the LINE CR1 family share the same 3′-end with tRNA-derived SINEs from the same genome [20]. Later other examples were found and to date many LINE/SINE pairs, sharing the same 3′-end sequence have been identified [21, 22], including plants [23, 24], fish [25–27], insects [28], and mammals [29].

It was proposed to divide all LINEs in two groups: the stringent type and the relaxed type [22]. Transposons of the stringent type recognize their own 3′-end whereas transposons of the relaxed type do not involve any specific recognition of the 3′-end except for the poly-A tail. Because L1/Alu pairs do not share the same 3′-end sequence, the mammalian L1s were ascribed to the relaxed type. It has been suggested that the poly-A tail serves for RNA recognition and for the efficient L1-mediated retrotransposition [10, 18, 23, 30–32]. The direct monitoring of Alu retrotransposition in the human cells confirmed the requirement of a poly-A tail for Alu retrotransposition [18]. The phenomenon of poly-A tail expansion of new Alu insertions, presumably due to the slippage of the L1 ORF2 protein, was demonstrated in [33]. It was suggested that this effect may play an important role in maintaining activity of Alu elements [32]. It was experimentally confirmed that the poly-A tail is essential for L1 retrotransposition [14]. Recognition of poly-A tail by LINE retrotransposition machinery could explain formation of the processed pseudogenes [34], however many processed pseudogenes, lacking poly-A tails and derived from nearly all types of RNA, were found in various genomes [35, 36].

Co-localization of L1 RNA, ORF1 and ORF2 suggests that upon translation of both ORFs from L1 RNA they immediately form ribonucleoprotein particle (RNP) [37]. Additionally, it was discovered that L1 RT and RNA binding does take place and that it occurs at or near poly-A tail [37]. Branched molecules consisting of junctions between transposon 3′-end cDNA and the target DNA, as well as specific positioning of L1 RNA within ORF2 protein, were detected during initial stages of L1 retrotransposition in vitro [38]. Poly-A deletion did not have strong effect on retrotransposition while deletion of more substantial part reduced the number of transcripts [38].

The idea that secondary or tertiary RNA structure shared by L1 and Alu could be responsible for recognition and binding of ORF2, possibly along with a poly-A tail, was proposed by Boeke [30]. An important observation that retrotransposition may proceed even without a poly-A tail [35, 36] suggests presence of other important elements, playing a role in recognition of RNA by retrotransposition machinery.

For the stringent-type LINEs one of the important elements is a stem-loop structure located at the end of the 3′UTR [23–29]. Currently there is ample experimental evidence from different species that the 3′UTR stem-loop is essential for retrotransposition: for LINE SART1 in silkworm [28], for LINE UnaL2 in eel [39], for LINE ZfL2-1 and ZfL2-2 in zebrafish [40], for SINE SmaI in salmon [41] and for LINE R2 in insects [42]. The presence of a stem-loop at the 3′UTR of LINE and SINE, which share the same 3′-end, was found for a much broader range of species and LINE families, including L1 in algae [23] and monocot plants [24], Tad1 in fungi [43, 44], L2 in fish [25–27], and RTE in mammals [29]. These findings raise a question whether ORF2 functionality to recognize a stem-loop structure at the 3′-end of a stringent LINE/SINE pair was evolutionarily preserved for the relaxed type LINEs, and for L1s in particular.

An evolutionary study of LINEs, based on the RT domain of ORF2 protein, estimated that non-LTR retrotransposons are as old as eukaryotes, and revealed 11 distinct clades showing strict vertical descendance with no sign of horizontal transfer [45]. However, horizontal transfer was reported for a minor fraction of LINE clades; these include transfer of jockey elements within Drosophila [46], Bov-B transfer from Squamata to the ancestor of Ruminantia [47, 48], insertion of additional C-terminal domain into ORF2 of insect R1 from plant viruses [49], and L1 transfer from humans to bacteria *Neisseria gonorrhoeae* [50]. Phylogenetic analysis made on the entire LINE ORF2 sequences, for which corresponding SINE partner is known [51], confirmed 11 clades reported by [45] and further enlarged the number of clades to 15 [51]. LINEs, which are known to share 3′-end sequences with SINEs, appeared to be enriched in L2, CR1, RTE and Ted1 clades. However, the dataset taken for analysis did not include plants and invertebrates, for which examples of SINE/LINE pairs were found too [22, 24, 52]. For plants, L1s, which share the same 3′-end sequence with SINE, were reported for green algae [23] and maize [24]. Phylogenetic analysis performed solely for the L1 clade and based on the entire ORF2 sequences, revealed monophyletic groups for green algae, land plants and vertebrates. Since land plants emerged from green algae, L1s of green algae show a strict mode of L1 recognition, and the strict-type L1s are observed in some plants, Ohshima proposed a model of parallel relaxation of stringent L1 RNA recognition in plants and mammals [51].

Evolutionary studies of L1 and Alu families in humans also presented evidence for the vertical evolution [53, 54]. Human L1 families were further subdivided into subfamilies, which were sequentially derived from a single lineage ending up in the currently active L1PA1 group of L1-Ta subfamily [53]. Analysis of Alu sequences led to identification of more than 200 families with 143 source elements [54].

Given that *(i)* evolution of LINEs in general and of L1s in particular showed mainly the vertical mode [21, 45, 53] with the stringent LINE/SINE pairs found among the different clades including L1; *(ii)* L1 RNA and ORF2 form stable RNP, and binding of the ORF2 to L1 RNA was reported to take place at or near poly-A tail [37, 38]; *(iii)* sequences, which lack poly-A tail, such as pseudogenes derived from different RNA genes, may undergo retrotransposition [35, 36], we suggest that L1 RNA recognition and binding with ORF2 could be evolutionarily preserved, though not at the sequence level but at the level of the RNA secondary structure. To test this hypothesis we investigated human L1 and Alu sequences for the presence of position-specific conserved stem-loop structures. We found highly conserved stem-loop position at the 3′UTR of L1 and at the 3′-end of Alu elements in human genome. Comparative analysis of this structure with other LINE 3′UTR stem-loop structures, which were experimentally shown to be essential for retrotransposition, revealed conservation of the structure without sequence homology. We found other conserved stem-loop positions at 5′UTR and at the end ORF2 proteins.

## Results

### L1

We performed an analysis of human L1 and Alu transposon sequences for the presence of position-specific stem-loop structures. Analyses were done for sets of presently active L1 transposons taken from [3], a set of 6622 L1s, divided into 27 subfamilies, as reported in [53] (the coordinates of all L1s used in this study are given in Additional file 1), and a set of 401 242 Alus, divided into 213 subfamilies as reported in [54] (coordinates of all Alus used in this study are given in Additional file 2). All transposon sequences were annotated with stem-loop structures, and this annotation was used for the construction of stem-loop coverage profiles (see Methods). High values in the stem-loop coverage profiles correspond to the conserved stem-loop positions.

**Fig. 1:**
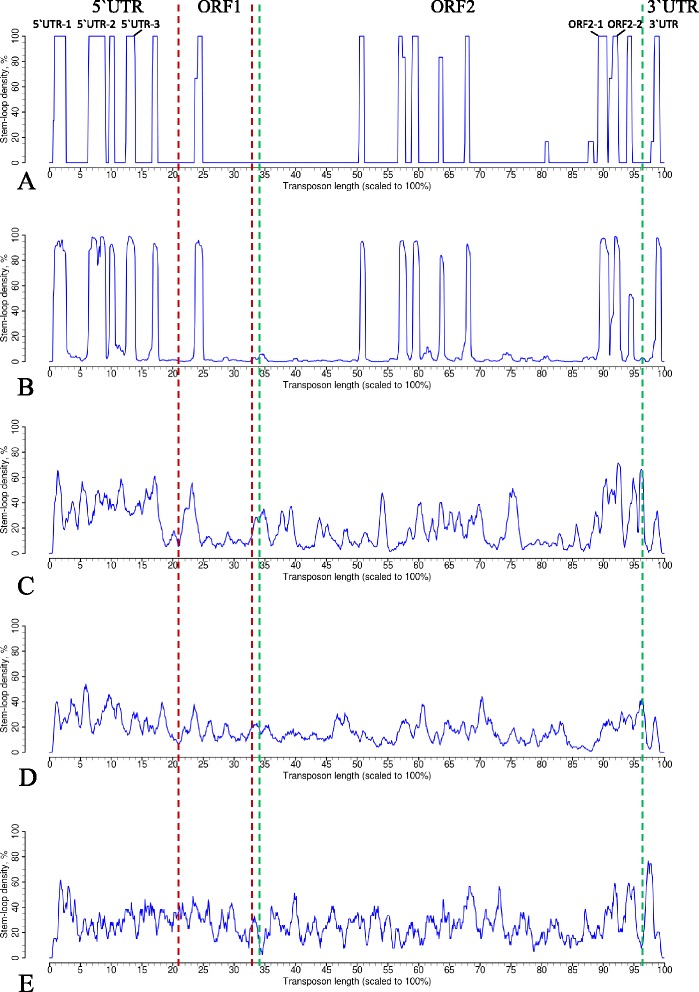
Stem-loop coverage profiles. **a** 6 hottest L1 transposons reported as active in [2]; **b** L1PA1, the most active L1 subfamily from [46]; **c** L1PA8, middle-aged subfamily from L1PA clade [46]; **d** L1PB1, subfamily from L1PB clade [46]; **e** L1MA1, subfamily from L1MA clade [46]

The stem-loop coverage profiles for different sets of L1 sequences are shown in Fig. 1. Figure 1a depicts conservation profiles for 6 most active L1 transposons taken from [3]; the profiles for the representative L1 family clades – L1PA, L1PB and L1MA – are presented in Fig. 1b-e. The conserved stem-loop positions of the most recent L1PA1 subfamily coincide with the stem-loop positions of 6 most active transposons. The level of position conservation gradually decreases with the age of L1 subfamily due to insertions, deletions and 5′UTR truncations, which affect transposon length, and also due to mutations affecting the stem-loop structure. The stem-loop conservation profiles for all 27 L1 subfamilies from [53] are provided in Additional file 3 in the order following the phylogenetic tree depicted in Fig. 2 in [53]. This trend of relaxation of position conservation is clearly seen in the direction from younger to older families and is in agreement with L1 vertical evolution. The profiles of 6 most active L1s and of the enlarged set of 33 active L1s, also reported in [3] are almost identical (Additional file 4).

**Fig. 2:**
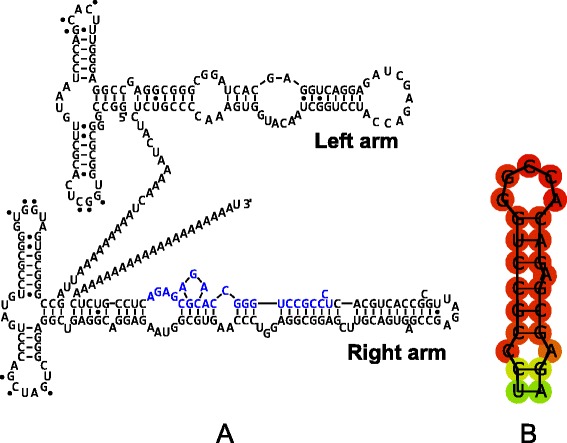
Alu and 3′-end stem-loop secondary structure for AluYa5 active element. **a** Alu secondary structure. Alu is composed from two monomers of 7SL RNA. Each monomer has two short stem-loops connected with U-turn at the 5′-domain and a long stem-loop with bulges at the 3′-domain. 3′-end sequence that can form stem-loop is highlighted in blue. **b** Alu 3′-end stem-loop structure from the sequence highlighted in *blue* in the section (A)

Reconstructed stem-loop conservation profiles revealed presence of conserved positions along the entire transposon length at 5′UTR, ORF2 and 3′UTR. According to the profiles of currently active L1s and L1PA1 subfamily, we distinguished three characteristic regions at 5′UTR (5′UTR-1, 5′UTR-2 and 5′UTR-3), two characteristic positions at the end of ORF2 (ORF2-1 and ORF2-2) and, importantly, a conserved position at 3′ UTR. All these positions are discussed below.

Although phylogenetic analysis of L1 ORFs sequences shows evolution of a strictly vertical type, this is not the case for non-coding parts of L1, 3′UTR and 5′UTR, which are not conserved at the sequence level. L1 changed 5′UTR several times in the course of evolution [53]. We analyzed 5′UTR regions of groups of L1 subfamilies having one type of 5′UTR as it was proposed in [53]. The stem-loop conservation profiles for 5′UTR regions for L1 subfamilies are presented in Additional file 5. The youngest group includes L1PA subfamilies from 1 to 8, and the profile reflects four conserved positions along 5′UTR region. As expected, significantly less conservation is observed for the older groups.

The coordinates of stem-loops along the entire transposon length for the consensus sequence of each L1 subfamily are given in Additional file 6. We also checked how the mutations, which lead to the corrupted ORFs affect stem-loop structures. The stem-loop profiles of L1s with intact and corrupted ORFs are given in Additional file 7. At least half of the mutations affecting ORFs also affect stem-loop structures in the ORF1 and ORF2 regions. However 3′UTR region remained the most conserved in terms of stem-loop structure conservation compared to 5′UTR region.

### Alu

The RNA secondary structure of Alu monomers is thought to be the same as that of 7SL RNA since Alu is composed of two 7SL Alu-domains [17]. Each monomer has two short stem loops connected with U-turn at 5′-domain and a long stem-loop with bulges at 3′-domain [17] (Fig. 2 depicts active AluYa5 element). Mutagenic experiments with Alu monomer binding affinity to SRP9/14 showed that retrotransposition is affected more by the mutations in the binding region of the left monomer, while the mutations in the binding region of the right monomer do not have a strong effect on retrotransposition. This finding suggests different roles two monomers may play in retrotransposition. Most likely, while the left Alu monomer is bound to SRP9/14, the right monomer participates in the recruitment of LINE RT, of which process the stem-loop recognition could be an important step. 7SL RNA structure contains three stem-loops, two in 5′-domain and one in 3′-domain, and each potentially could be used in binding with LINE RT. However the reverse transcription starts precisely from the 3′-end of Alu, and that is why the structures, close to 3′-end could be essential for binding with the RT.

Here we performed an analysis of 401 242 Alu elements, divided into 213 subfamilies, as reported in [54], for the presence of a 3′-end stem-loop structure. For all the elements from all families, AluJ, AluS and AluY, we detected a potential stem-loop structure, located at the very end of 3′-end region, several nucleotides before the poly-A tail. However, if the right Alu monomer accepts the known 7SL RNA structure, the predicted 3′-end stem-loop is hidden and its entire palindromic sequence is a part of the stem of the right arm (the sequence highlighted in blue in Fig. 2b). The structure of this stem-loop is given in Fig. 2b and it has a 5 bp central loop together with an internal symmetrical loop (1–1) located at a distance of 6 bp from the central loop.

**Fig. 3:**
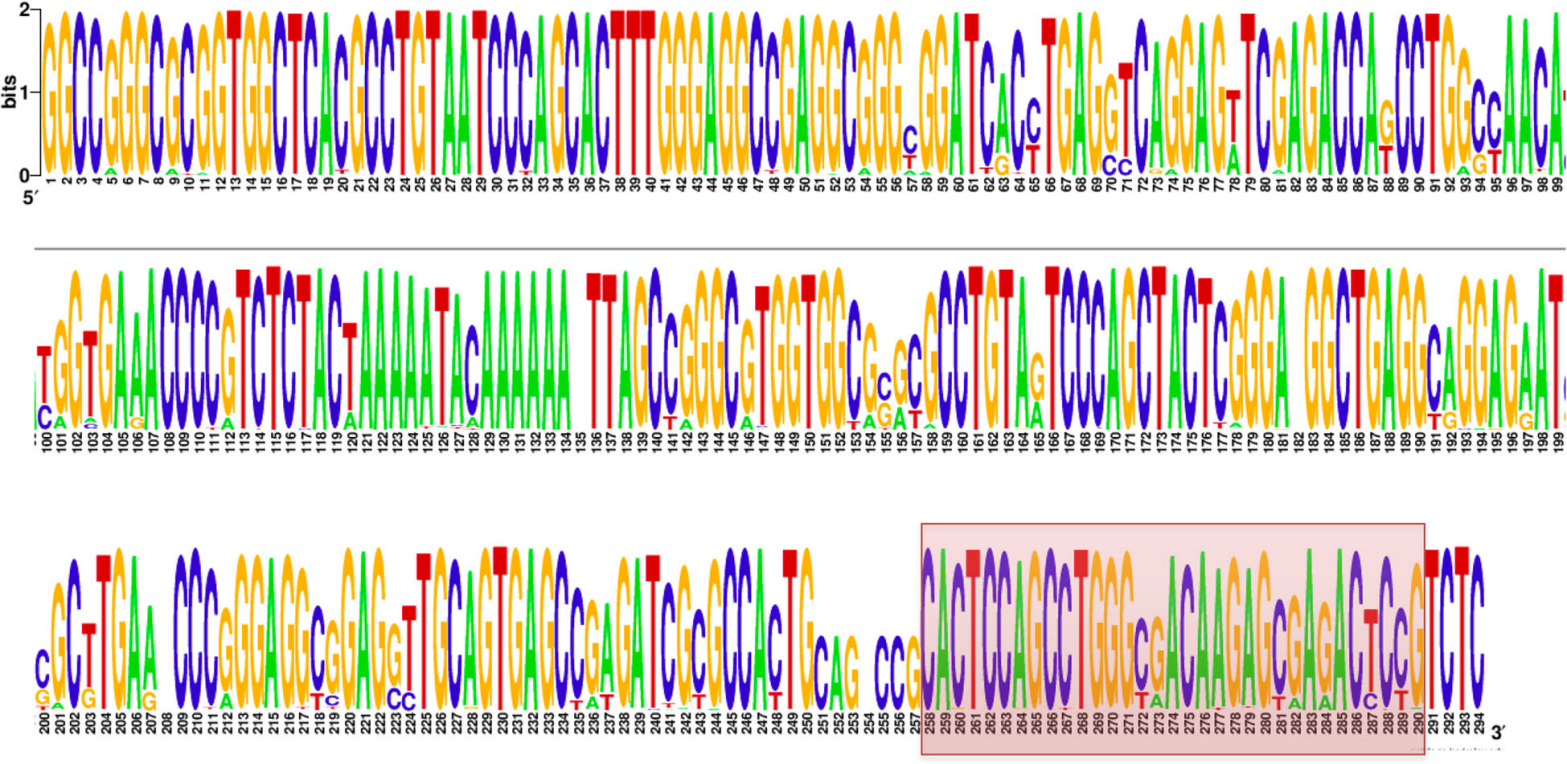
Alu sequence logo profile constructed from consensus sequences of 213 Alu families. Position of 3′-end stem-loop structure is highlighted in *red*

We took consensus sequences for each of 213 Alu subfamilies, constructed multiple alignment and built a sequence logo profile (see Fig. 3). The level of sequence conservation for the left Alu monomer is higher than that for the right. The position of 3′-end stem-loop structure, which reveals some degree of variation, is highlighted in red. The small region of 7 bp to the left of the stem-loop with a low conservation is because of the insertion into two highly active Alu elements, AluYB8 and AluYB9 [55]. The coordinates of 3′-end stem-loop structures for the consensus sequences of each subfamily is provided in Additional file 8. We analyzed structural features of the predicted stem-loops depending on the subfamilies and found the following trend. All of the predicted stem-loops fall into two major classes – those with a bulge and those without a bulge. Almost all stem-loops from the ancient families, AluJ, have a 3′-end stem-loop without a bulge, while AluY contains almost all 3′-end stem-loops with a bulge, similar to those presented in Fig. 2. Proportions of stem-loops with and without a bulge in the middle-aged AluS family are almost equal. A possible role of a bulge in stem-loop structures in general is discussed below.

### LINE 3′ UTR stem-loop

**Fig. 4:**
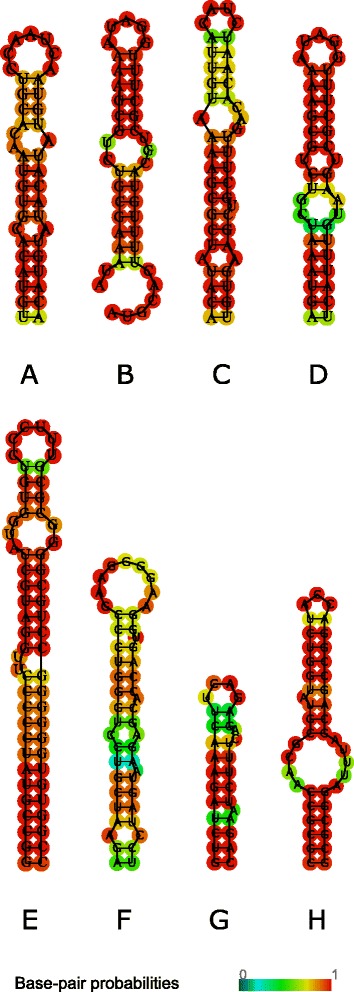
RNA secondary structures for 3′UTR stem-loops from different species. **a** L1 3′UTR stem-loop from human genome taken from 6 hottest L1 transposons reported as active in [2]. **b** L2 3′UTR stem-loop from eel experimentally reported as recognized by LINE-encoded RT [26]. **c**-**d** two L2 3′UTR stem-loops from zebrafish (ZfL2-1 and ZfL2-2) experimentally reported as recognized by LINE-encoded RT [40]. **e** R1 3′UTR stem-loop from silkworm experimentally reported as recognized by LINE-encoded RT [28]. **f** L1 3′UTR stem-loop from rat [49]. **g** L1 3′UTR stem-loop from maize (Malik, Burke et al. [45]). **h** R1 3′UTR stem-loop from mosquito (Malik, Burke et al. [45])

3′ UTR region occupies ~200-245 bp at the end of the L1 transposon immediately after ORF2. Stem-loop density profiles (Fig. 1) revealed that active transposons and the youngest L1PA1 family have a distinct peak in the 3′UTR region. For different sets of active L1 elements we extracted sequences corresponding to 3′UTR stem-loops and reconstructed their secondary structures. For two sets of experimentally confirmed active transposons from [3] the corresponding stem-loop is located within the last 50 bp of L1. 3′UTR stem-loop secondary structure is presented in Fig. 4a.

Then we extracted 3′UTR stem-loop structures for different classes of LINEs L1 3′UTR stem-loop from 6 hottest human L1 transposons reported as active in [3] (Fig. 4a); L2 3′UTR stem-loop from eel [26] (Fig. 4b); two L2 3′UTR stem-loops from zebrafish (ZfL2-1 and ZfL2-2) [40] (Fig. 4c-d); R1 3′UTR stem-loop from silkworm experimentally reported as recognized by LINE-encoded RT [28] (Fig. 4e); L1 3′UTR stem-loop from rat [56] (Fig. 4f); L1 3′UTR stem-loop from maize [45] (Fig. 4g); and R1 3′UTR stem-loop from mosquito [45] (Fig. 4h). Four of them: L2 from eel (UnaL2) [26], L2 from zebrafish (ZfL2-1 and ZfL2-2) [40], and R1 from silkworm (SART1) [28] were experimentally reported as participating in RT binding. The characteristic feature of the reported stem-loops is an asymmetrical internal loop, or a bulge, located at a distance of 4–6 bp from the central loop (Fig. 4a-h).

We examined the structure of 3′UTR stem-loop located at the very end of 6 L1 human hot transposons and found that it has a structure with a bulge most similar to that of a zebrafish. By structural similarity we mean the length of the loop and position of a bulge with respect to the loop. We also reconstructed secondary structures for 3′UTR stem-loops from the set of 33 active L1s and 6622 L1s. The characteristic stem-loop with a bulge was found in all 33 active L1s and in more than 50% of 6622 L1s. In other cases, the structure represented a long stem-loop up to 11–12 bp with one unpaired base at one side of the stem at the position of the bulge, and in some cases with 5 up 10 asymmetrically unpaired bases in the bulge.

**Fig. 5:**
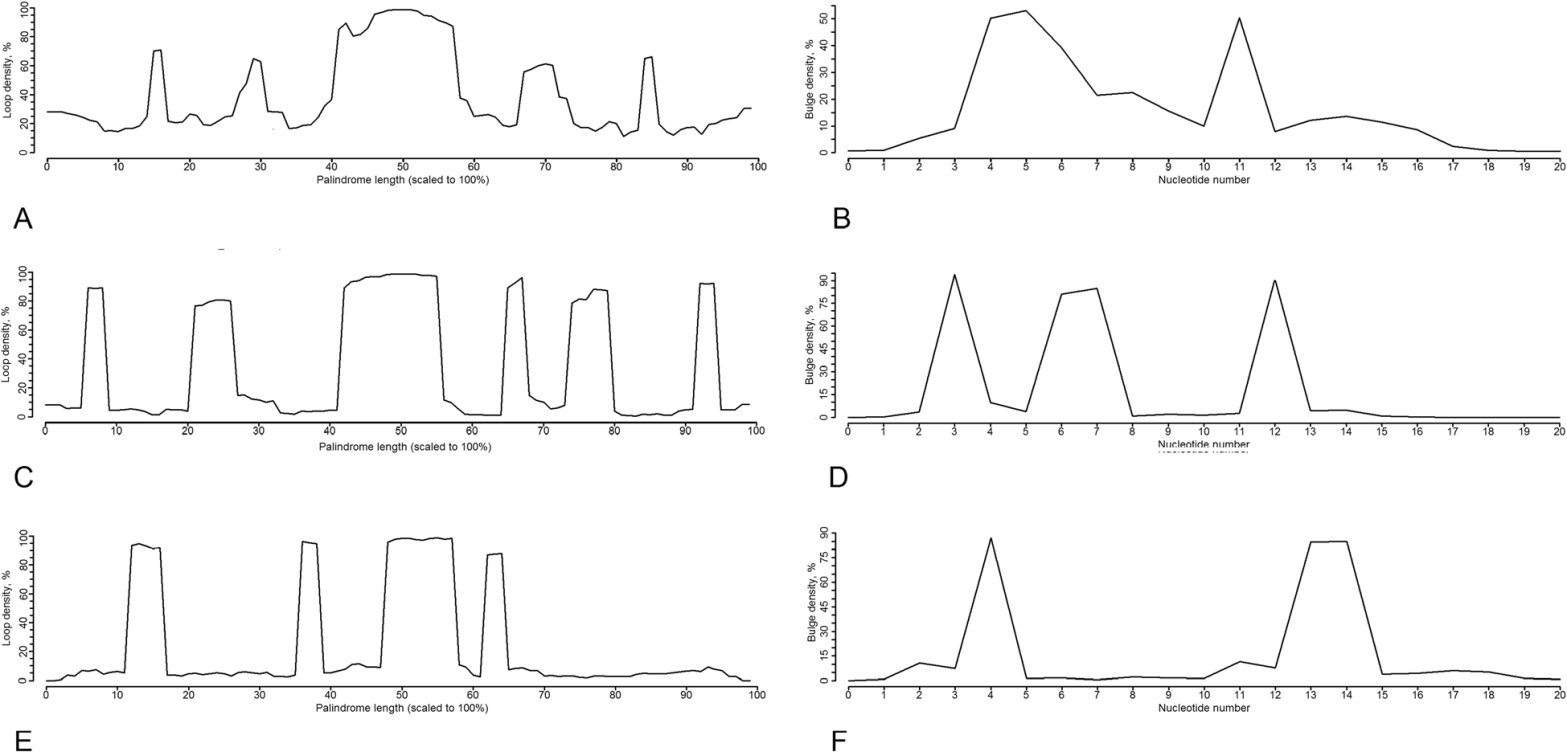
Pairing-unpairing profiles for L1 3′UTR and 3′-end Alu stem-loops. The values of the resulted profile reflect how many stem-loop structures will have unpaired bases at a given position. Pairing-unpairing profiles for (**a**) L1 3′UTR stem-loop normalized to 100% length and (**b**) L1 3′UTR stem-loop, stem only; (**c**) stem-loop at the end of the Alu right arm normalized to 100% length and (**d**) stem-loop at the end of the Alu right arm, stem only; (**e**) Alu 3′-end stem-loop normalized to 100% length and (**f**) Alu 3′-end stem-loop, stem only

To assess the distribution of the bulge size and position in L1 and Alu 3′-end stem-loops we constructed pairing/unpairing profiles (see Methods) where non-zero values correspond to the unpaired bases (Fig. 5). The profiles for L1 (Fig. 5a-b) show the presence of two internal loops, when the first internal loop occupies region from 4–5 bp if counting from the central loop. For Alu we compared two structures – one that is formed by the 3′-end, and the second one within the last 50 bp of the 3′-end. The profile of the 3′-end stem-loop (Fig. 5c-d) revealed the presence of three unpaired regions located at 3 bp, 6–7 bp and 12 bp from the central loop. For the stem-loop corresponding to the end of the right arm single unpaired nucleotides are located at 4 bp and the bulge positions are 13–14 bp (Fig. 5e-f). The profiles showed that the L1 3′-end stem-loop profile is more similar to the 3′-end Alu stem-loop rather than to the stem-loop at the end of the right arm.

### LINE ORF2 stem-loop binding region

The currently accepted model of LINE RNA recognition by ORF2 is that there are two types of recognition – the stringent and the relaxed. In the stringent type RT recognizes its own 3′UTR tail, and in the relaxed type RT does not require any specific recognition except for the poly-A tail. Division into the stringent and the relaxed type came from the observation that some LINE/SINE pairs share the same 3′-end. For the stringent type, the experimental studies showed that a 3′UTR stem-loop is required for retrotransposition.

Evolutionary studies of LINEs for which corresponding SINE partners are known showed that in the phylogenetic tree, constructed from ORF2, the stringent- and the relaxed-type LINEs are intermixed, and the stringent-type LINEs are present in almost all branches including mammals, fishes, insects and plants (see phylogenetic trees in [21, 51]).

**Fig. 6:**
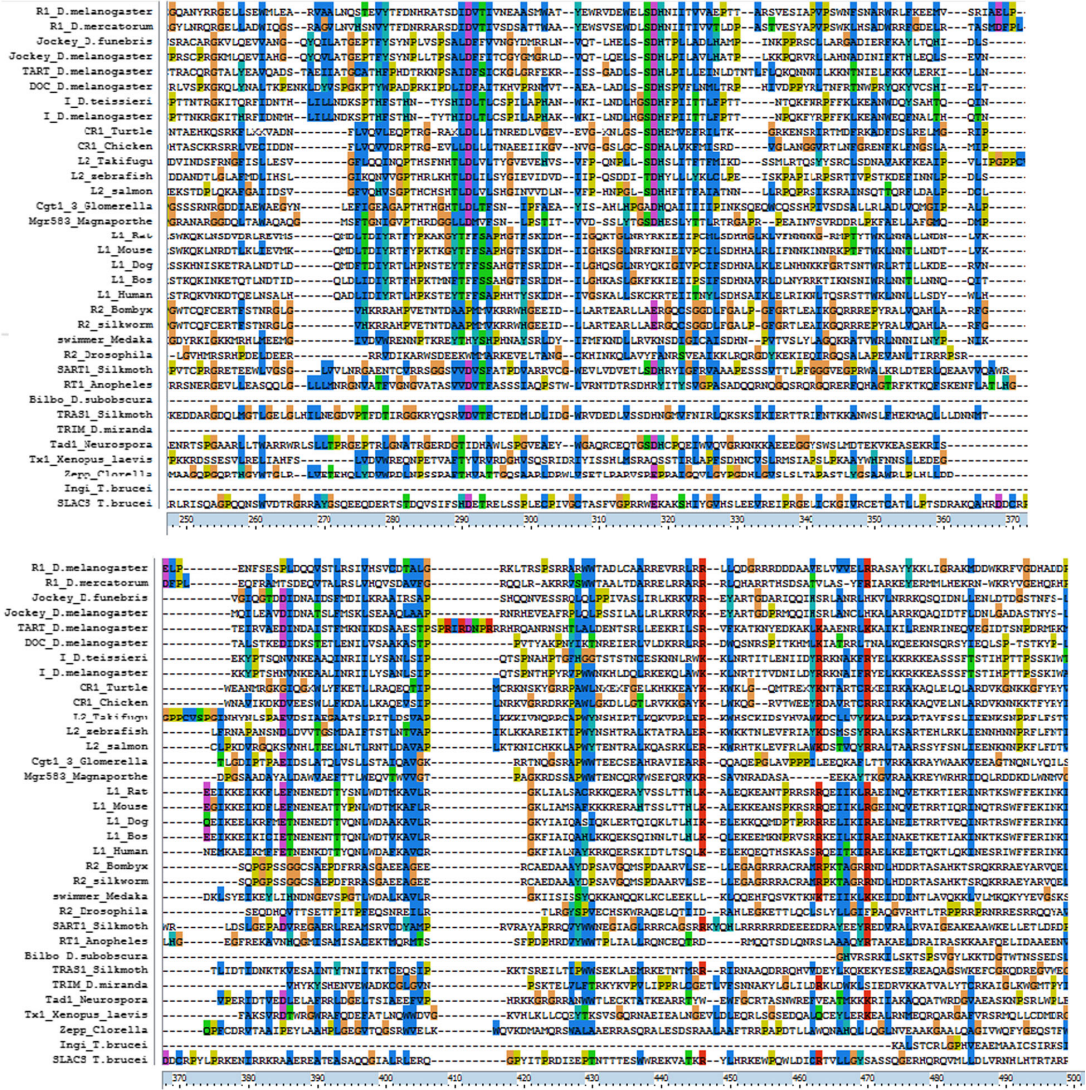
Alignment of ORF2 region between EN and RT domains (250–500 aa)

It was shown that for zebrafish LINEs ZfL2-1 and ZfL2-2, belonging to the stringent type L2 clade, the region of the ORF2 that binds to the 3′-end lies between the endonuclease and reverse transcriptase domains [40]. Here we took ORF2 sequences from different LINE clades and investigated the region between EN and RT domains for the amino-acid conservation – approximately the region between 250 aa and 500 aa. The region of 250–500 aa of ORF2 alignment is presented in Fig. 6. Alignment is done for different types of LINE, it is not limited to L1 and includes L2, R1, R2, CR1, I, Jockey, and others. Although the level of conservation of the region between EN and RT domains is lower compared to the EN and RT domains (see full alignment in Additional file 9), it is still noticeable that the sequences are homologous. Therefore they could retain the function to recognize stem-loops. It is not excluded that 3′-end stem-loop recognition was conserved throughout evolution for all types of LINEs, both the stringent and the relaxed type.

### 5′ UTR and ORF2 stem-loops

**Fig. 7:**
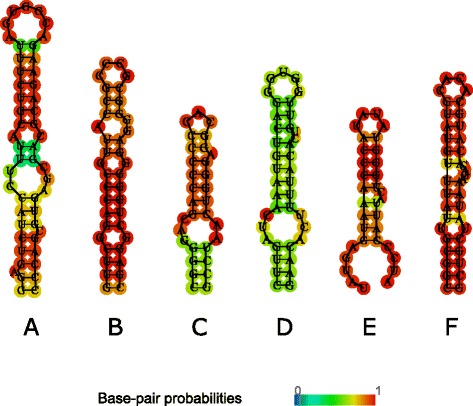
RNA secondary structures of human L1 stem-loops from 5′UTR and ORF2 conserved positions. All the positions are highlighted in the L1 coverage profiles in Fig. 1. **a** 5′UTR-1 region, corresponds to ~50-100 bp. **b** 5′UTR-2 region, correspond to ~420-600 bp. **c** 5′UTR-3 region, correspond to ~600-840 bp. **d**-**e** ORF2-1 region, corresponds to 5397–5437 bp. **f** ORF2-2 region, corresponds to 5540–5583 bp

The L1 stem-loop coverage profiles revealed three conserved stem-loop positions at 5′ UTR region, which we designated as 5′UTR-1, 5′UTR-2 and 5′UTR-3 (Fig. 1). The first conserved position at 5′UTR-1 corresponds to the very beginning of the transposon (~50-100 bp in the transposon coordinates). The stem-loop structure from the active transposons from this region is very stable, with a stem of 20 bp (Fig. 7a). It also has an internal asymmetrical loop (3–2) located at a distance of 10 bp from the central loop. The second conserved position (5′UTR-2) corresponds to the transposon region of ~420-600 bp. The structure located in this region is GC-rich and has a long stem of 17 bp. Its characteristic feature is a stretch of four C in the loop (Fig. 7b). The 5′UTR-3 region (around 600–840 bp) also contains GC-rich stem-loop structure with a stretch of 5 G-C pairs in the upper stem and 3 G-C pairs in the lower stem (Fig. 7c).

Two other stem-loop conserved positions are located at the end of ORF2-encoded sequence. The characteristic feature of these structures is that they contain short repeat sequences in the central loops. The region ORF2-1 (5397–5437 bp) contains two stem-loop structures. The first (5504–5536 bp) has poly (G) (4–5 G) in the central loop (Fig. 7d). The second ORF2-1 stem-loop contains TATA (or TATATA) repeat (Fig. 7e). The stem-loop structure from the ORF2-2 region (5540–5583 bp) is a long 16-bp structure with a bulge located at a distance of 6 bp from the central loop (Fig. 7f). The central loop contains CACA repeat.

## Discussion

Retrotransposition is a multistage process that includes transcription, formation of ribonucleoprotein particles, translation, posttranslational modifications, transport back to the nucleus and integration in the DNA by the mechanism termed Target Primed Reverse Transcription (TPRT) [57]. Each stage involves transposon RNA interactions with different proteins including those encoded by the transposon itself, and often stem-loop structures participate in binding.

### 3′UTR. Stringent versus relaxed

The 3′UTR region was shown to play a crucial role in recognition of the stringent-type LINE RNA by ORF2 protein [40, 42, 58]. A stem-loop located at the very end of 3′UTR was confirmed to be an essential recognition motif for the stringent-type LINE-SINE pairs.

The LINE retrotransposon of insects, R2, requires 250 nucleotides at 3′UTR for recognition of its own RNA [42]. The R2 ORF2 protein from a silkworm *Bombyx mori* can identify 3′-end not only of its own RNA but also of R2 of *Drosophila melanogaster*. It is noteworthy that sequence similarity of this region in *Bombyx mori* and *Drosophila melanogaster* R2 is very low. Secondary structure models of 3′ UTR were predicted for both organisms and it appears that 3′UTR region contains several hairpins [59]. Later it was confirmed that for silkworm SART1 the central 3′UTR stem-loop is essential for retrotransposition, and that the transcription starts mostly from several telomeric repeat-like GGUU sequences just downstream of this stem-loop [28].

For eel genome it has been demonstrated that UnaL2 (LINE) and UnaSINE1 (SINE) share a similar 3′ tail, which is necessary for the successful UnaL2 transposition [26]. Moreover UnaL2 RT can recognize 3′ tail of UnaSINE1, thereby allowing its mobilization. The conserved 3′ tail consists of two parts: the stem-loop (a GGAUA loop) and the pentanucleotide repeat ([TGTAA]*n*). Both of them are necessary for the transposition of UnaL2. It has been suggested that 5′ part of the GGAUA loop is recognized by the RT [60]. The stem includes a small internal loop, which contains a single unpaired cytidine and U-U mismatch. Both are crucial for the successful transposition. This internal loop may contribute to the flexibility of the entire stem, which can be required for UnaL2 function [39].

In zebrafish ZfL2 ORF2 protein binds the hairpin located at 3′ tail via specific site between EN and RT domains [40]. Specifically, it was found that recognition can be bipartite, involving general recognition of the stem and specific recognition of the loop.

In salmon genome SINE SmaI element is derived from tRNA, and its 5′ region forms a tRNA-like cloverleaf structure while the 3′ domain forms an extended stem-loop structure, wherein the loop region is believed to be recognized by the LINE encoded RT [41]. It was also demonstrated that three other salmon SINEs, SlmI, HpaI and OS-SINE1 share the same 3′-end tail as LINE RSg-1 [27].

In turtle genome, 3′-end sequences of SINE family, designated as the tortoise PolIII/SINE, are also almost identical to 3′-end of LINEs from CR1 family [20]. This CR1-like LINE family is widespread in birds and in many other reptiles. Examples, in which 3′-ends of tRNA-derived SINEs are derived from 3′-ends of LINEs include transposons from turtles, fish, mammals and plants [22].

Homology between 3′-end of Vhc SINEs of bivalve mollusks and LINEs from CR1 clade of mollusk *Crassostrea gigas* was reported in [61]. Predictions of secondary structures of several Vhs SINEs from different mollusk species revealed the presence of 3′UTR stem-loops, however no experimental evidence is available to confirm the role of a 3′-end stem-loop in invertebrates. Another stem-loop from the central 40-bp subdomain of the V-domain was found to be conserved in SINEs in mollusks and arthropods, but its function remains unknown.

The experimentally confirmed evidence that a stem-loop structure at 3′UTR region, shared between LINE/SINE pairs, plays an important role in LINE ORF2 recognition raises a question about applicability of a similar type of recognition for the relaxed LINE-SINE pairs. A phylogenetic analysis of ORF2 sequences showed the strict vertical evolution of this protein. It was shown that for zebrafish LINEs, the region recognizing 3′-end stem-loop lies between the EN and RT domains [40]. We showed that this region shows homology across different taxa and different LINE families. Importantly, the stringent LINE/SINE pairs are intermixed with the relaxed type across all branches of the phylogenetic tree starting from algae and protists, and are present in almost all divisions: in plants, insects, fish, reptiles and mammals.

We presented evidence that the recent human L1 and Alu families, to which the currently active retrotransposons belong, have a stem-loop structure at 3′-end of their sequences. We also identified presence of a 3′-end stem-loop structure in all (~400 000) analyzed human Alu sequences, however the formation of this structure *in vivo* is an open question. The tertiary structure of Alu is thought to be the same as that of two Alu domains of 7SL RNA bound by the RNA connector sequence. After Alu is transcribed it immediately forms ribonucleoprotein particle [62] together with the SRP9/14 heterodimer [55, 63], poly A-binding protein (PABP) [64] and perhaps some other proteins, which can bind RNA [19]. Crystal structures of SRP9/14 bound to the Alu domain revealed the exact positions of binding [65]. SRP9/14 binds strongly to the conserved core of 5′ domain, which forms a U-turn connecting two stem-loops (Fig. 1 in [65]). This part of 5′Alu-domain RNA is highly conserved in SRP RNAs from eubacteria to higher eukaryotes [66]. Mutagenesis experiments showed that the SRP binding regions of the two Alu monomers have different outcome, with the mutations in the right monomer having a minor effect on retrotransposition. This could mean that the right monomer can be used for RT recognition. While the left Alu-domain may be bound to SRP9/14, the protruding arm (positions 220–260) at 3′-domain of the right Alu monomer can potentially be used for RT recognition (Fig. 2). The structure of stem-loop at the end of the right arm (~220-260 bp) is structurally similar (in the sense that it also contains an internal bulge) to L1 3′UTR and zebrafish and eel LINE 3′UTR structures. For this structure to emerge, Alu right monomer has to acquire a conformation different from the accepted 7SL RNA. However, it is possible that the PBP bound to the polyA tail contributes to the unfolding of 3′domain, and this can lead to the formation of 3′-end stem-loop structure, that can be used for the recognition by the ORF2 protein.

### *Cis* preference versus *trans* complementation

A number of authors sought to study the effect of preference of LINE machinery to various cellular RNA, such as LINE and SINE RNA and mRNA [30, 34, 37, 58]. It was shown that L1 proteins predominantly retranspose their own RNA – the effect named as *cis* preference [58]. In the same study it was demonstrated that L1 encoded proteins can retrotranspose cellular RNA, though at the much lower frequencies of 0.01-0.05% compared to the retrotransposition frequencies of the wild-type L1 RNAs. The mechanism through which retrotransposition of the non-LINE RNA occurs is termed *trans* complementation. It is believed that *cis* preference does not assume RNA recognition, and protein-RNA binding occurs cotranslationally due to ORF2 and L1 RNA proximity [58] while *trans* complementation requires a protein-RNA recognition both for Alus and pseudogenes. However mechanisms of the recognition remain unclear.

Even with *cis* preference it was demonstrated that L1 RNA, ORF1 and ORF2 form the ribonucleoprotein particle, and the binding of L1 RT takes place at or near the L1 RNA poly-A tail [37]. In the work of [34] *cis* preference was tested on the mutated L1 RNA transcript with stop codons in both ORFs and on the non-mutated L1 RNA, and no difference in the retroposition frequency was detected. This result supports the hypothesis of specific L1 RNA recognition by the encoded proteins. Nevertheless *cis* versus *trans* preference was confirmed in this study too. It should be emphasized that the noticeable effect of *cis* preference, where the proximity can facilitate binding, does not exclude the direct recognition of L1 RNA by the L1 encoded proteins. The presence of the secondary structure that participates in the binding of the RT with the retrotransposed RNA does not contradict to the effect of *cis* prefenece and *trans* complementation where both proximity and binding specificity can play an essential role.

### Poly-A tail

Alu and L1 do not share the same 3′-end sequence except for the poly-A tail. It was believed that the poly-A tail is essential for the human Alu and L1 retrotransposition [30]. Direct monitoring of Alu retrotransposition in human cells revealed the functional importance of this element in Alu retroposition [18]. Poly-A tail is a part of Alu gene and not a result of polyadenylation, because Alus are transcribed by Pol III. Deletion of the poly-A tail from the DNA template almost abolished Alu transposition diminishing frequency by 1000 times [18]. An interesting observation of Alu tail expansion after the round of retroposition was reported in [33]. Expansion is thought to occur due to the slippage of L1 RT during the reverse transcription, and this effect could have provided evolutionary advantage to Alu elements to maintain their activity by counteracting to the natural loss of A-tail [32]. Another finding of the same study was that the TPRT priming was not random with the priming positions being identified at least 25 bp from 5′-end of the poly-A tail [33]. This points to the constraints, which can be imposed by the bound proteins. Poly-A binding protein (PBP) can also bind Alu, and it was demonstrated that this protein is associated with SINE RNP [67].

The requirement of poly-A for the L1 retroposition was demonstrated in the work of [14]. Experiments were done with an engineered L1/MALAT RNA where L1 polyadenylation signal was replaced with 3′-end of the long non-coding RNA MALAT1, which can form a triple helical structure followed by a tRNA-like sequence. The triple helix can prevent RNA degradation in the absence of the poly-A tail [68]. L1/MALAT RNA lacking the poly-A tail is translated, but is not retrotransposed in *cis*. Addition of 16 or 40 poly-A sequence restored retrotransposition in *cis*. Also it was shown that poly-A tract is required for an association of the ORF2 and the retrotransposition-defective L1.

Whether the poly-A tail recognition is done involving the sequence directly or via the PBP remains an open question. Right after the translation of ORF1 and ORF2 both proteins associate with L1 and form RNP that goes back to the nucleus [69, 70]. It was found that PBP is associated with L1 ribonucleoprotein complex and is essential for retrotransposition [71]. Moreover, specific positioning of L1 RNA with ORF2 protein was observed [38]. Non-specific interaction of LINE RNA and ORF2 in human cells was found between the 180 aa carboxy-terminal segment (CTS) of L1 ORF2 protein and the human L1 RNA [72]. It was shown that a newly translated L1 ORF2 protein immediately binds with a high affinity to a native template with its C-terminal tail.

Some studies do not support the idea that poly-A tail is essential for retroposition. In the study of [38] the authors demonstrated *in vitro* that poly-A deletion did not have a strong effect on retrotransposition, while deletion of a more substantial 3′UTR region noticeably reduced the number of transcripts [38]. The abundance of retropseudogenes without poly-A tail also supports the idea that poly-A tail recognition can be bypassed in retrotransposition [35, 36]). These are tRNA-related tailless retropseudogenes, usually composed of 5′-part of the original tRNA or SINE founder RNA. Hundreds of thousands of tailless retropseudogenes derived from nearly all types of RNAs were discovered [35]. It was shown that L1-ribonucleoprotein particles are enriched in pseudogene transcripts [31]. These findings tell about the possibility for retrotransposition machinery to work without the poly-A tail, and for another essential recognition motif to be present in the retrotransposed sequences. This motif can be an RNA secondary structure. Here we hypothesize that an Alu element can mimic L1 with a structurally similar stem-loop though further experiments are needed to support this hypothesis.

### Experimental cassettes studying retrotransposition

Assays to study retrotransposition are based on the idea that the indicators inserted in the cassettes will be expressed only upon a successful round of retrotransposition. Usually the indicators are inserted into 3′ UTR sequences. For example, *mneoI* cassette consists of the neo gene in the reverse orientation of the L1 transcription, inserted into 3′UTR, and the entire LINE-1 expression vector ends with SV40 polyadenylation signal [73]. This signal forms secondary structures, which were shown to be functionally important [74]. Specifically, a stem-loop structure, located downstream of the poly-A sequence correlates with the cleavage intensity [75]. Besides, a stem-loop structure was artificially added to the upstream region of *neo* gene in order to ensure binding of bacteriophage MS2 coat protein for the detection of L1 cellular localization by fluorescent in situ hybridization (FISH) [73].

Similarly, MALAT1, used in the experiments of [14] and disrupting the 3′UTR region, ends with a tRNA-like sequence, which is capable to form stem-loop structures. Thus, in all assays studying retrotransposition, 3′UTR region is disrupted by the insertions of various sequences, but all these sequences end with stem-loop structures.

### Stem-loop structure and a bulge

Consensus structure of 3′-end transposon has an internal asymmetrical loop, or a bulge. Experiments with mutants from eel 3′-UTR stem-loop showed that the deletion of the bulge completely blocks the transposon activity [26]. Earlier it was shown for introns and viruses that the internal bulge in the stem of a stem-loop structure could be functionally important. For example, domain 5 (D5) of group II introns has a stem-loop structure with an internal bulge in the stem. It was shown that the loop, bulge and trinucleotide region in the lower stem are conserved features of splicing machinery of group II introns and also of spliceosome [76]. Specifically, the loop, the bulge and the triplet may bind essential metal ions to position functional groups participating in catalysis [76]. In HIV-1 the internal bulge of a stem-loop plays an important role in packaging through mechanisms, which are not fully understood [77]. Internal loops play an important role in discriminating between miRNA precursors and other conserved hairpins [78]. Other examples where internal loops of stem-loop structures participate in binding include the murine IgM [79] and the yeast ribosomal protein L30 [80]. The study of thermodynamics for the reaction of a set of DNA hairpins containing internal loops showed that the size of an internal loop does matter and all targeting reactions proceed with negative changes in free energy, indicating that reactions proceed spontaneously [81].

For the detected 3′UTR stem-loop structure with a bulge we found that the position of a bulge is conserved in all active L1 transposons reported in [3] and conserved for more than 50% of the analyzed 6622 L1 elements from 27 families reported in [53]. Taking into account the experiments with eel transposons where deletion of a bulge completely abolished transposition, as well as other evidence of bulges playing an essential role, we hypothesize that the presence and location of a bulge in 3′UTR stem-loops plays an important role in RT recognition.

### 5′UTR

L1 transcription starts from its internal promoter. L1 5′UTR region contains internal promoter for two L1 ORFs, and it is not conserved at the sequence level. The length of the 5′UTR region is around 1000–1200 bp. Here we made analysis and present coordinates of the 6 active hottest transposons from [3]. First 100 bp were shown to possess promoter activity [5]. A binding site for the transcription factor YY1 was identified at positions ~3-26 bp with the core element sequence AAGATGGCC (~11-19 bp) [82]. The other binding sites (472–477 bp and 572–577 bp) for SRY family transcription factors were also identified [83]. Other transcription factors belonging to the family RUNX were shown to bind to 83–101 bp in 5′UTR region [84].

We found three conserved positions for stem-loops in 5′UTR regions of L1 active transposons belonging to L1P1 family. The first highly conserved 5′UTR-1 stem-loop (50–100 bp) is located within the 100 bp region reported as having the promoter activity, and it does not overlap with the binding sites reported for YY1 transcription factor, which are located in the first 50 bp area. The position of 5′UTR-2 stem-loop (423-461 bp) immediately precedes the SRY family binding site (472–477 bp), and there is a stem-loop (534-567 bp) located right before the second reported transcription factor binding site (572–577 bp).

Stem-loop structures in promoter regions with an important functional role were reported mostly for viruses [85, 86]. Perfect palindromes with the stem length of 5 bp were found in TATA-less promoters of ~ 5% of human genes [87]. Comprehensive analysis of five potential promoters of the HNRNPK gene showed that the one containing a palindrome [88] showed the highest activity. Further experimental efforts are required to study the role of stem-loop structures located at 5′UTR regions of the transposable elements.

### ORF2

Stem-loop structures found at the end of ORF2 gene have characteristic dinucleotide repeats TATA and CACA, and also poly (G) sequence in the central loop, suggesting a possible role these stem-loops may play in the recognition by specific proteins. The role of the CACA-repeat as a regulator of the mammalian alternative splicing was revealed in [89]. Dinucleotide repeat motifs were found to be enriched in enhancers in Drosophila [90]. It is possible that position-specific stem-loops at the end of ORF2 protein play a role in the formation of ribonucleoprotein complexes, which direct the transport of L1 RNA into specific cell locations. Little is known about mRNA structures *in vivo.* The recent study of mRNA structures revealed abundance of intra-molecular double-stranded RNA [91]. Moreover, depletion of the coding regions and enrichment of 3′UTR was observed. These results confirm the important role of RNA secondary structures in the post-transcriptional pathways of mRNAs, but further experiments are required to elucidate their function.

## Conclusions

Here we presented an evidence for the presence of a highly conserved 3′UTR stem-loop structure in L1 and Alu transposons in human genome. We demonstrated that this 3′UTR stem-loop of L1 transposons is structurally similar to 3′UTR stem-loops of other LINEs from different species, which were experimentally reported as playing an essential role in retrotransposition [26, 28, 40], specifically RNA-binding region of ORF2 were determined in [26, 28, 40]. The region that binds to the 3′UTR stem-loop in zebrafish ZfL2-1 and ZfL2-2 transposons, shows homology across various LINEs from a wide range of species. The latter suggests that the functionality to recognize a stem-loop structure at 3′-end may have persevered through evolution for relaxed LINE/SINE pairs, including L1/Alu pair. Here we hypothesize that the binding of both L1 and Alu RNAs with L1 RT can be done via the structurally similar stem-loop structure at 3′-end of the transposon RNA. The other conserved stem-loop positions at 5′UTR and at the end of ORF2 suggest their possible functions in the protein-RNA interactions, but to date no experimental evidence has been reported.

## Methods

### Sets of L1 and Alu transposons

A set of 6622 L1 full-length elements divided into 27 subfamilies was taken from [53]. Consensus sequences for each subfamily were also taken from [53]. The sets of currently active L1 were composed from transposons experimentally reported as active in [3]. One set is composed from 33 reported active transposons, while the other set is made from a subset of 6 most active transposons, the activity of which is 10 times higher compared to other active elements [3].

A set of 401 242 Alus was taken from [54]. A division into 213 subfamilies and corresponding consensus sequences was based on the same study [54].

### *DNA Punctuation*, a program that searches for stem-loop structures

A stem-loop structure is a palindromic structure that consists of a double-stranded stem, which is formed by completely or partially complimentary sequences, and a single-stranded loop. We define the following search parameters: minimum stem length (*minStemLen*), maximum stem length (*maxStemLen*), maximum loop length (*maxLoopLen*), and maximum number of mismatches in stems, which include gaps (*maxMismatch*). The task can be formulated as follows: given the sequence, find the longest complementary substrings in the range of *minStemLen, maxStemLen*, separated by a distance less than *maxLoopLen*, with no more gaps or mismatshes than *maxMismatch*.

The proposed algorithm is a dynamic programming algorithm based on the well-known Needleman–Wunsch sequence alignment algorithm [92]. However instead of looking for the alignment that produces a maximum score, we will look for the alignment with a minimum penalty score for gaps and mismatches. To work only with positive numbers we add nothing for matches and penalize with 1 for mismatches and gaps.

Thus, a matrix M, of size [(*minStemLen* +1) x (*minStemLen* +1)] is built as follows.

For 2 ≤ i ≤ *minStemLen* +1, 2 ≤ j ≤ *minStemLen* +1:

1 $$ \mathrm{M}\left(\mathrm{i},\mathrm{j}\right)= \min \left|\begin{array}{c}\hfill \mathrm{M}\left(\mathrm{i}\hbox{-} 1,\mathrm{j}\hbox{-} 1\right)+\mathrm{S}\left(\mathrm{i},\mathrm{j}\right)\left(\mathrm{mismatch}\ \mathrm{or}\ \mathrm{match}\right)\hfill \\ {}\hfill \mathrm{M}\left(\mathrm{i},\mathrm{j}\hbox{-} 1\right)+\mathrm{w}\left(\mathrm{gap}\ \mathrm{in}\ \mathrm{sequence}\ 1\right)\hfill \\ {}\hfill \mathrm{M}\left(\mathrm{i}\hbox{-} 1,\mathrm{j}\right)+\mathrm{w}\Big(\mathrm{gap}\ \mathrm{in}\ \mathrm{sequence}\ 2\hfill \end{array}\right. $$

Where S (i,j) = 0 (match), S (i,j) = 1 (mismatch), w = 1 (gap penalty).

In the context of stems, a match corresponds to complementary nucleotides, while a mismatch refers to non-complimentary nucleotides. The first column and the first row are filled by substring coordinates. The values in the first column correspond to the coordinates of the first substring, and the values in the first row correspond to the coordinates of the second substring.

For example, for the two sequences TACG and AATGC, the initialized matrix is:

After initialization the matrix is gradually filled according to the scheme (1). For example, M [2, 2] = min (M [1, 1] + 0, M [1, 2] + 1,M [1, 2] + 1) = min (0,2,2) = 0.

After the matrix is filled the program searches for the cell with a maximum minimum of its coordinates – max_ij_(min(i,j)), and whose value does not exceed *maxMismatch* – M(i,j) < = *maxMismatch*. If the corresponding coordinate is no less than *minStemLen*, the cell will be a starting point for the traceback processing. Let *maxMismatch* =2, and *minStemLen* =2, then the program searches for a maximum coordinate that has a value less or equal to 2. In the example this is the lowest right cell. The traceback processing is performed similar to the Needleman-Wunch algorithm with the only difference that the path is chosen according to minimum matrix values. The resulting stem structure is:

To avoid cases where sequence pairing in stems starts with a gap or mismatch we add an additional requirement of M (1,1) = M (2,2).

Source code in C and the program *DNA Punctuation*, implementing the aforementioned algorithm, is available at www.dnapunctuation.org.

### Stem-loop annotation and coverage profiles

Stem-loop structures were annotated with the program *DNA Punctuation* described above. We searched for structures with the stem length in the range of 15–50 bp, loop up to 10 bp, with 7 mismatches or gaps allowed. The stem-loop coverage profiles were constructed along the entire transposon length so that 1 is added if a base pair in the sequence is covered with a stem-loop and 0 otherwise. The total value for a given position was divided by the number of transposons, so that y-axis reflects a percentage of sequences having a base pair covered with the stem-loop. To adjust to a different length of the transposons we scaled the sequences to the normalized length of 100% with an average of 6 kb for L1 and 300 bp for Alu. In summary, the stem-loop coverage values reflect the percentage of analyzed sequences having sequence positions covered with stem-loop structures.

### Structure analysis

Secondary structures for the selected stem-loop sequences were reconstructed and visualized with RNA fold software (Vienna RNA Package 2.1.9) [93].

To investigate position conservation for a bulge or an internal loop for a set of stem-loops we constructed pairing-unpairing profiles for a set of position-specific stem-loop sequences. If a base is unpaired then we add 1 to the profile or 0 otherwise. The values of the resulting profile reflect how many stem-loop structures will have unpaired bases at a given position. Two types of profiles were constructed: one with the length of a selected stem-loop normalized to 100%, and the other for a stem where only x-coordinates correspond to the exact position in a number of bases starting from the loop (x = 0) and towards the base of the stem.

## Additional files

Additional file 1: Coordinates of 6622 L1s used in the study. (CSV 212 kb)
Additional file 2: Coordinates of 401242 Alus used in the study. (CSV 12945 kb)
Additional file 3: Stem-loop coverage profiles for 27 L1 subfamilies from (Khan, Smit et al. [53]) (PDF 2803 kb)
Additional file 4: Stem-loop profiles for active and highly conserved L1 transposons: (A) set of 6 hottest L1 transposons reported as active in (Brouha, Schustak et al. [3]); (B) set of 33 active L1 transposons reported as active in (Brouha, Schustak et al. [3]); (C) set of 6622 highly conserved L1 transposons (see Methods for selection criteria). (PDF 221 kb)
Additional file 5: Stem-loop profiles for 5′UTR regions of groups of L1 subfamilies having one type of 5′UTR as it was proposed in (Khan, Smit et al. [53]). (PDF 703 kb)
Additional file 6: Coordinates of stem-loops along the entire transposon length for the consensus sequence of each L1 subfamily. (CSV 472 kb)
Additional file 7: Stem-loop profiles for intact or non-intact ORFs: (A) set of L1 transposons with intact ORFs; (B) set of L1 transposons with frameshift mutations in ORFs. (PDF 217 kb)
Additional file 8: Coordinates of the 3′-end stem-loop for consensus sequences for 213 Alu subfamilies. (CSV 28 kb)
Additional file 9: Full alignment of ORF2 protein taken from different types of LINE, it is not limited to L1 and includes L2, R1, R2, CR1, I, Jockey, and others. (PNG 1346 kb)

## Abbreviations

3′UTR: 3′ untranslated region
5′UTR: 5′ untranslated region
EN: Endonuclease
FISH: Fluorescent in situ hybridization
LINE: Long interspersed element
ORF: Open reading frame
RNP: Ribonucleoprotein particle
RT: Reverse transcriptase
SINE: Short interspersed element
TPRT: Target primed reverse transcription
