# Supplementary material for: Conserved 3′ UTR stem-loop structure in L1 and Alu transposons in human genome: possible role in retrotransposition
Source: BMC Genomics. 2016 Dec 3;17:992. doi: 10.1186/s12864-016-3344-4 (PMC5135761; doi:10.1186/s12864-016-3344-4)

L1PA1

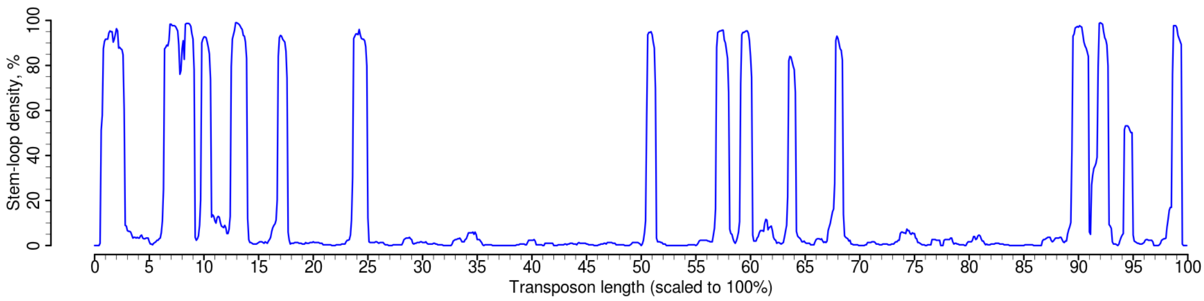

L1PA2

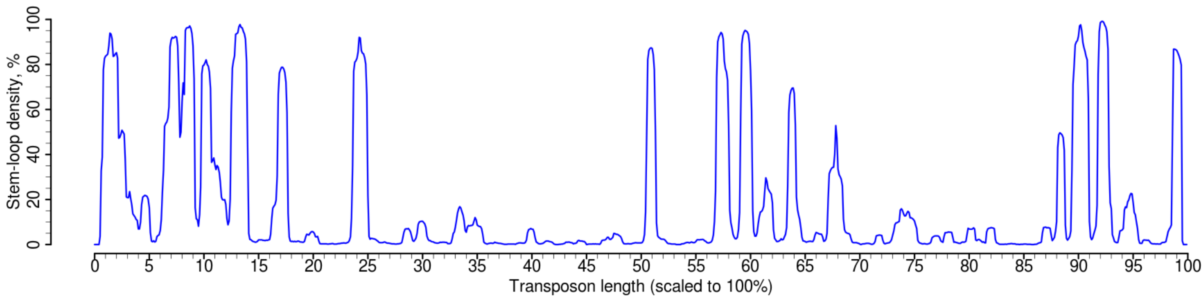

L1PA3

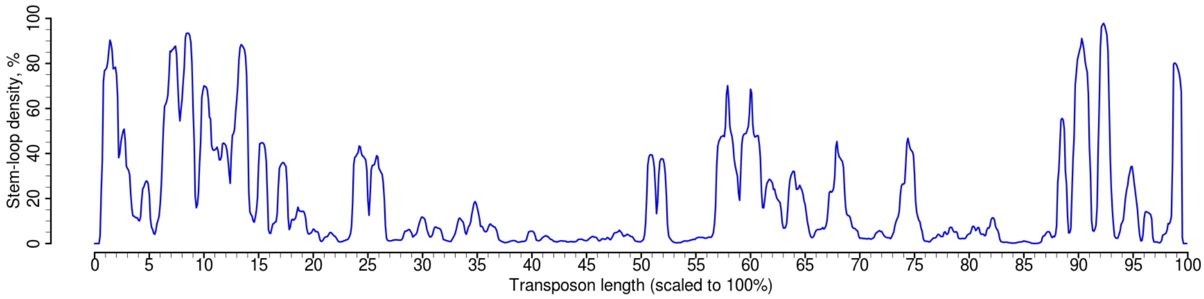

L1PA4

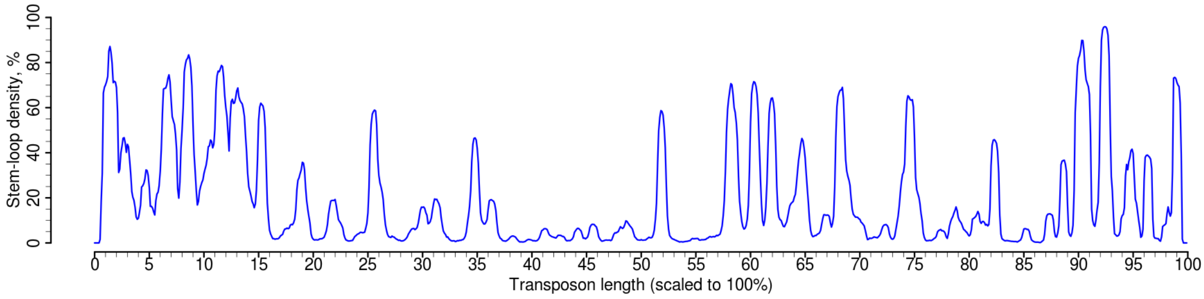

L1PA5

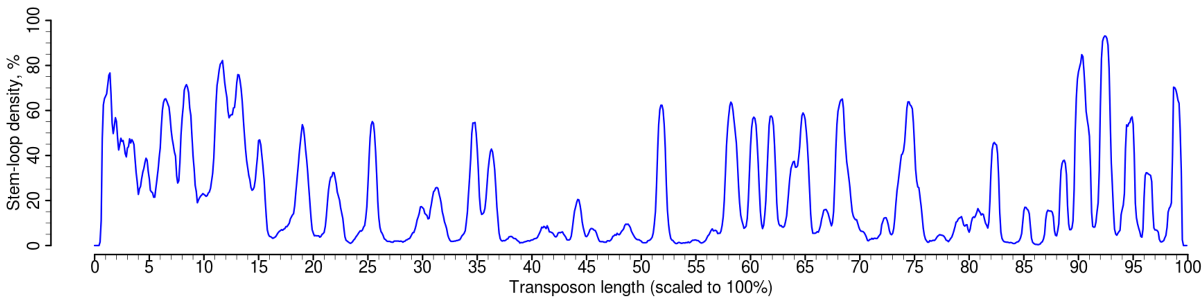

L1PA6

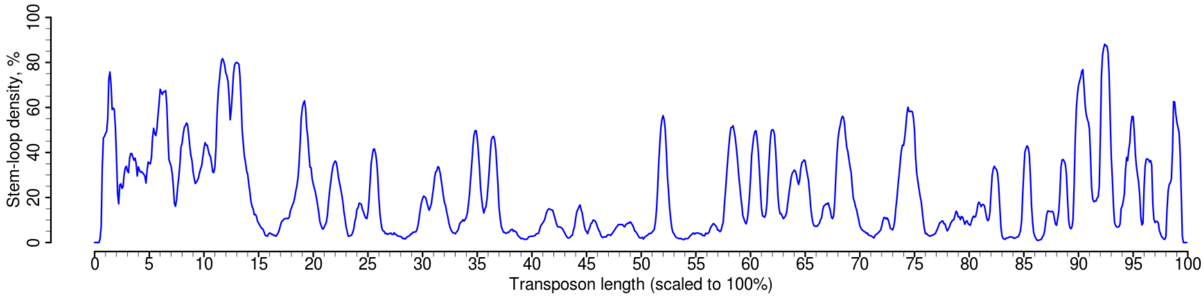

L1PA7

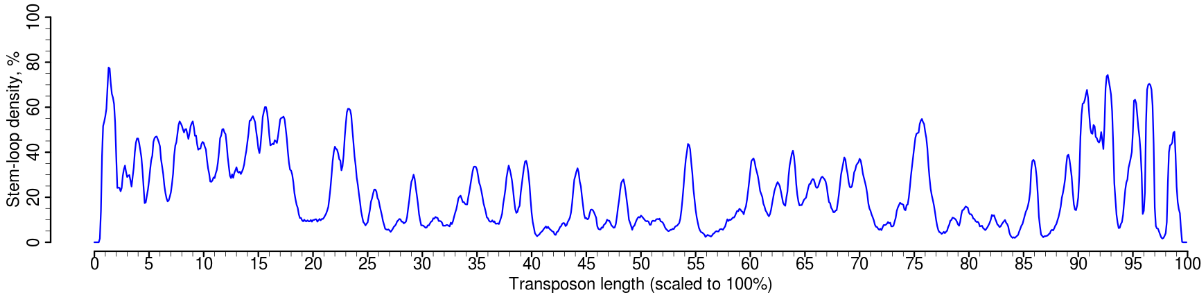

L1PA8

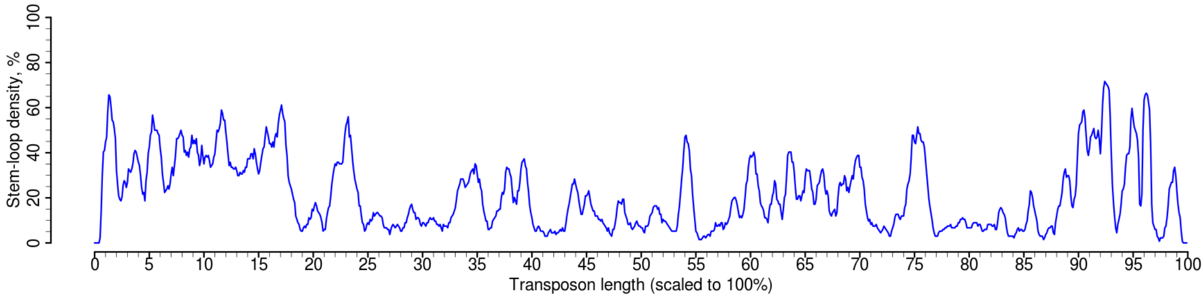

L1PA8A

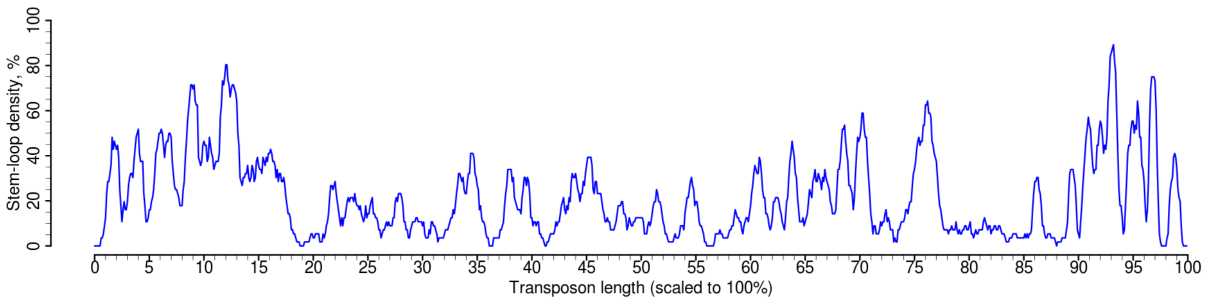

L1PA10

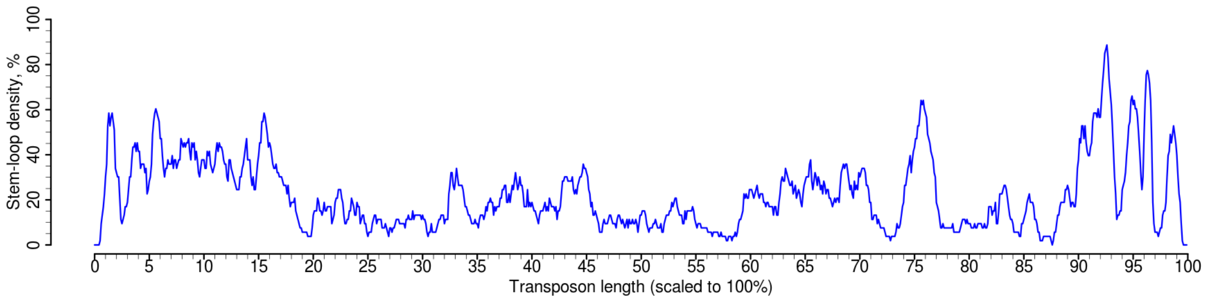

L1PA11

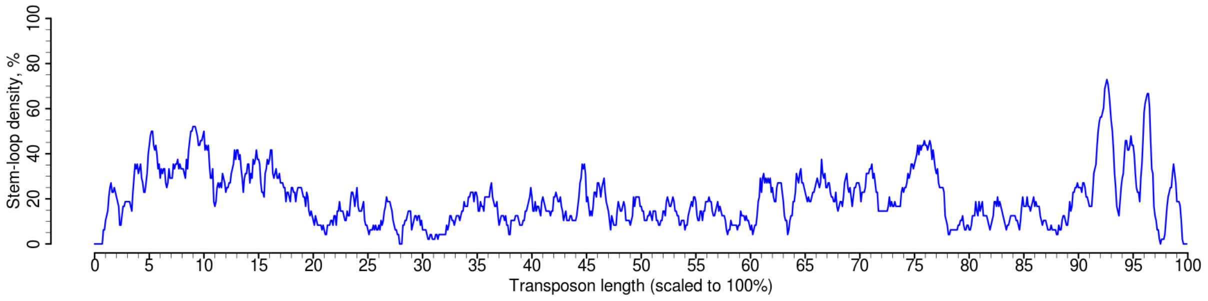

L1PA12

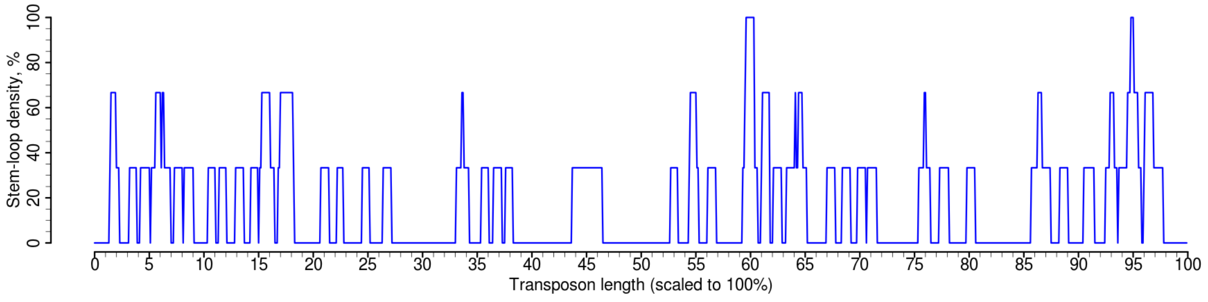

L1PA13A

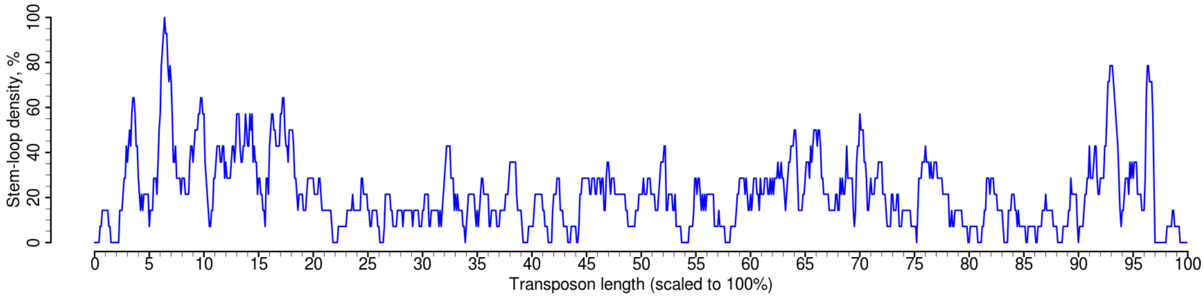

L1PA13B

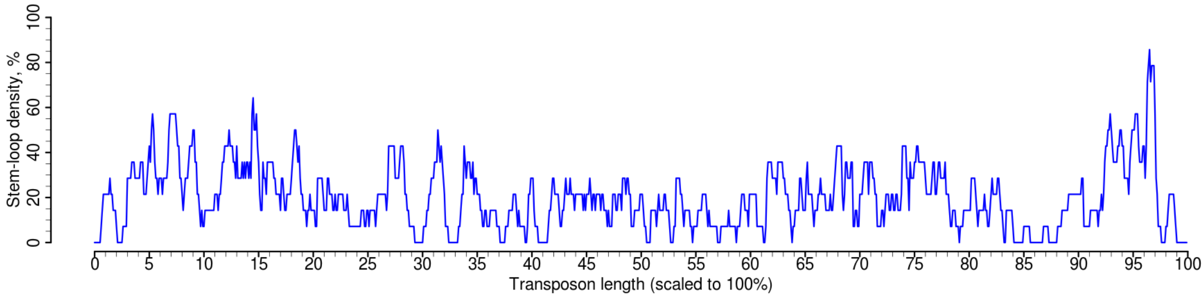

L1PA14

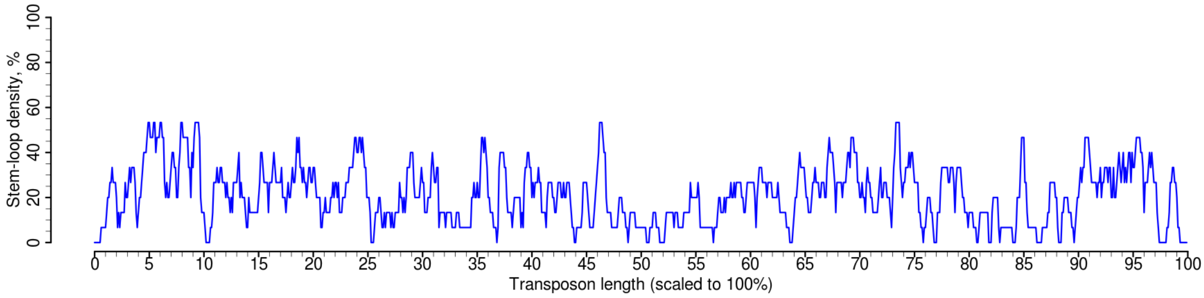

L1PA15

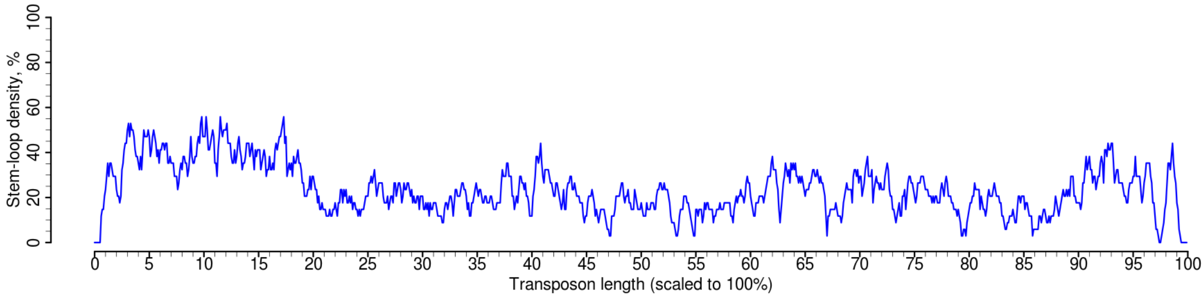

L1PA16

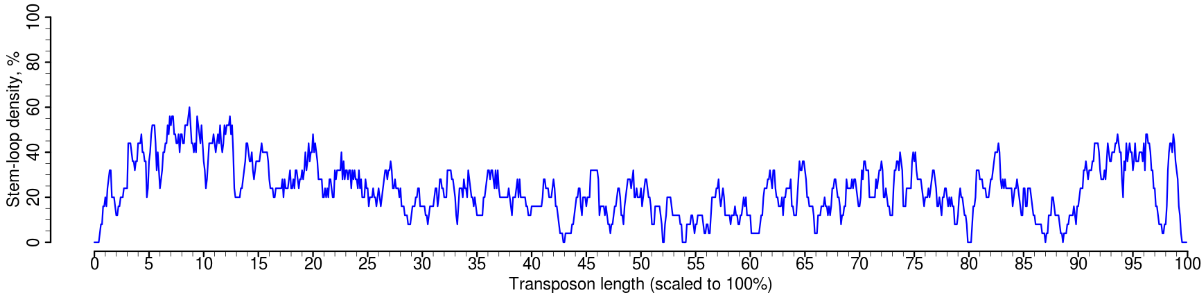

L1PA17

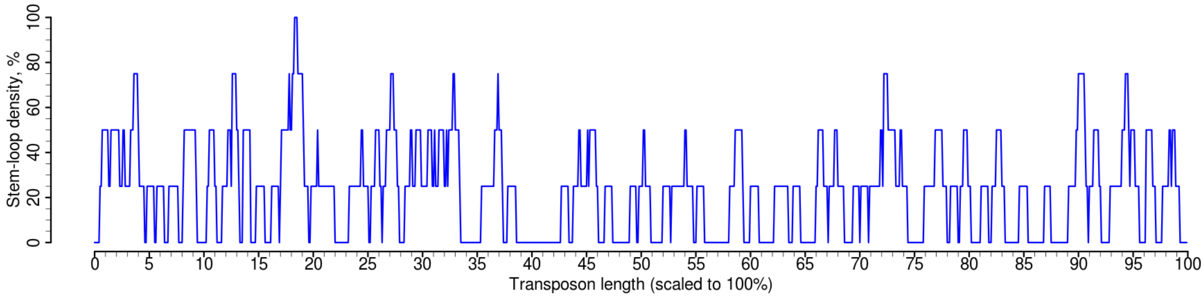

L1PB1

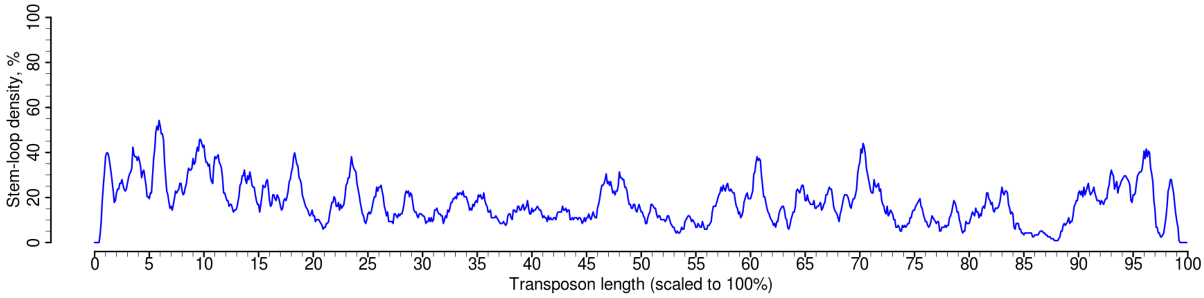

L1PB2

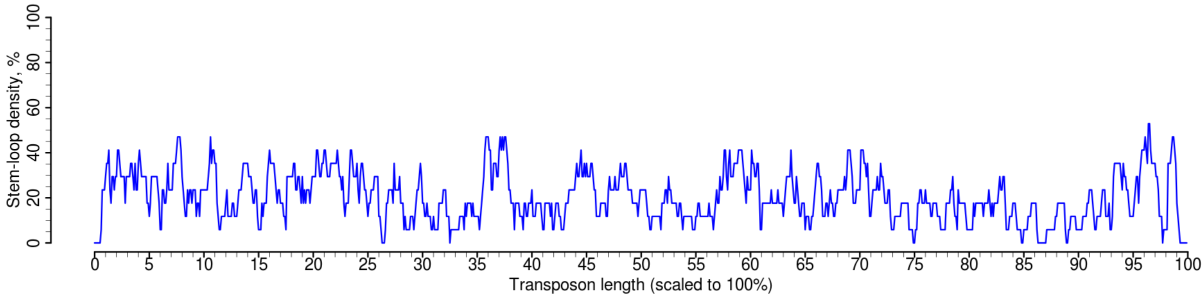

L1PB3

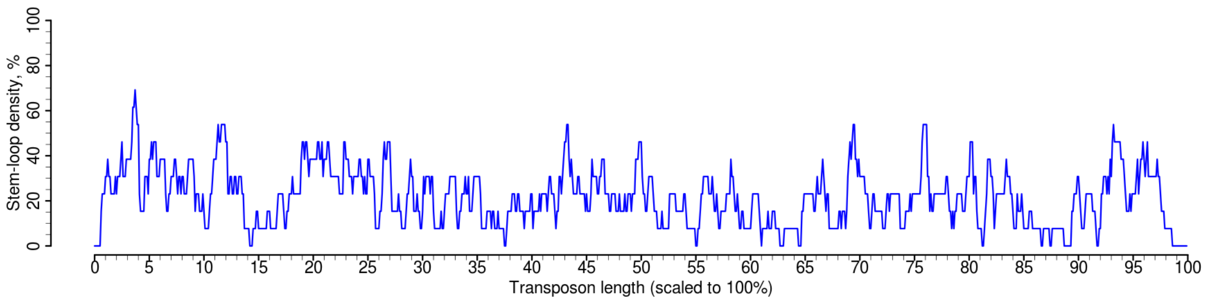

L1PB4

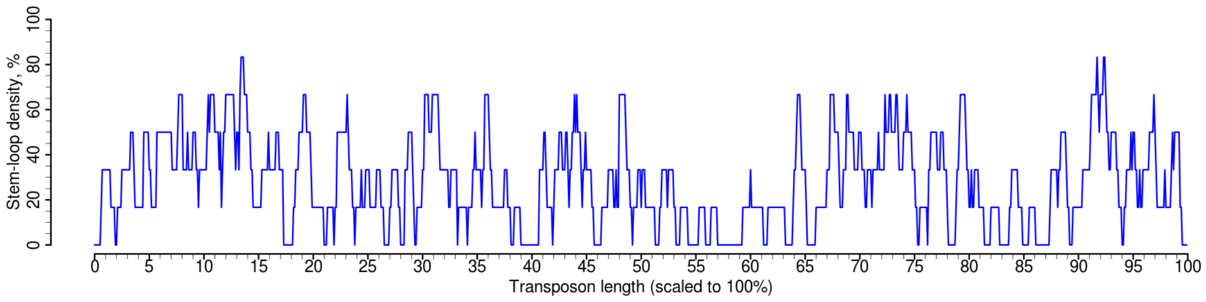

L1MA1

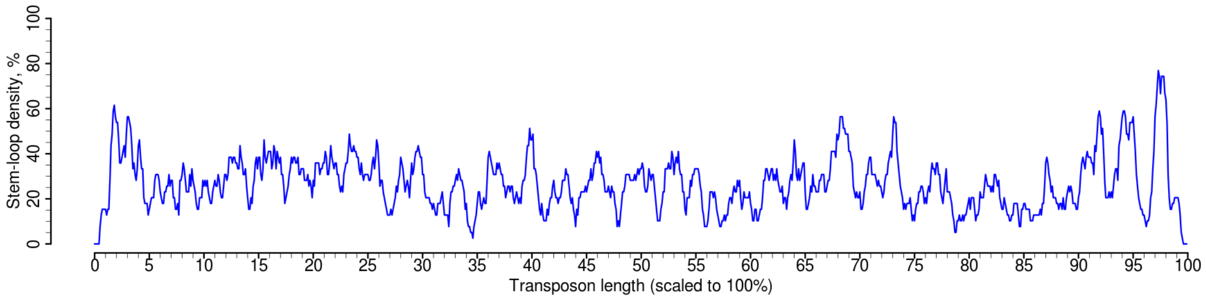

L1MA2

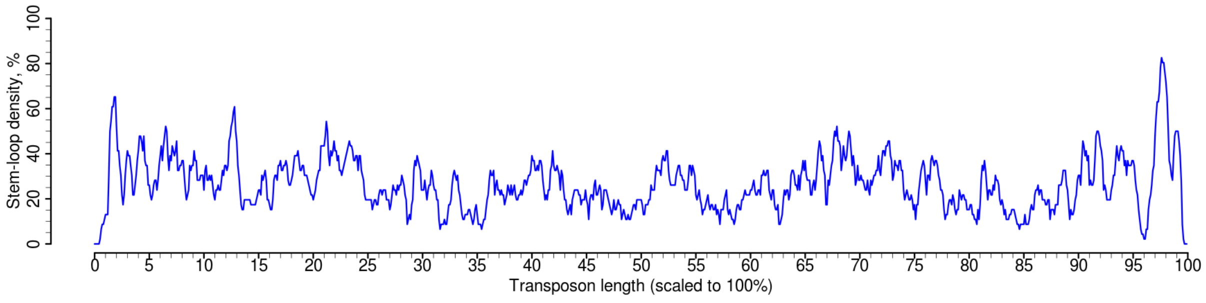

L1MA3

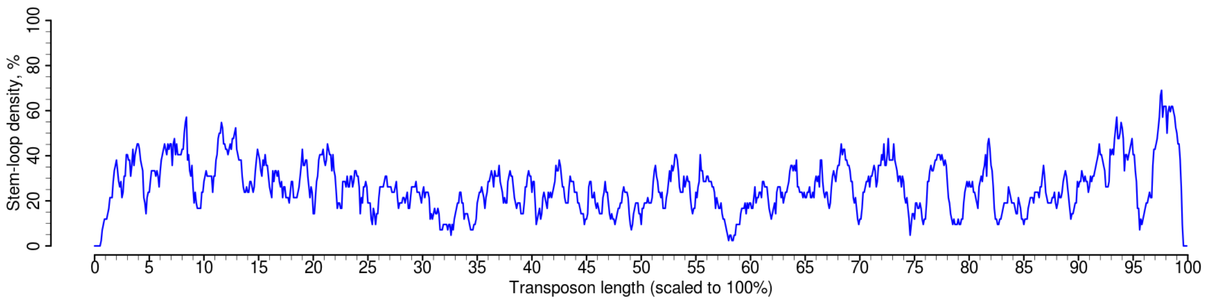

L1MA4

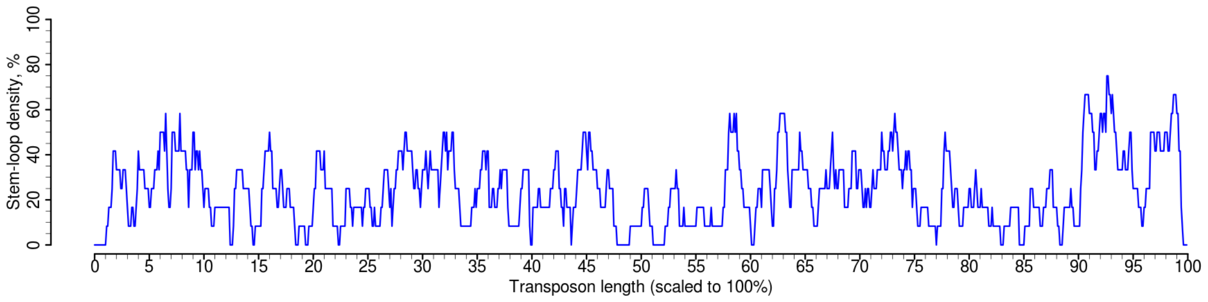

L1MA5

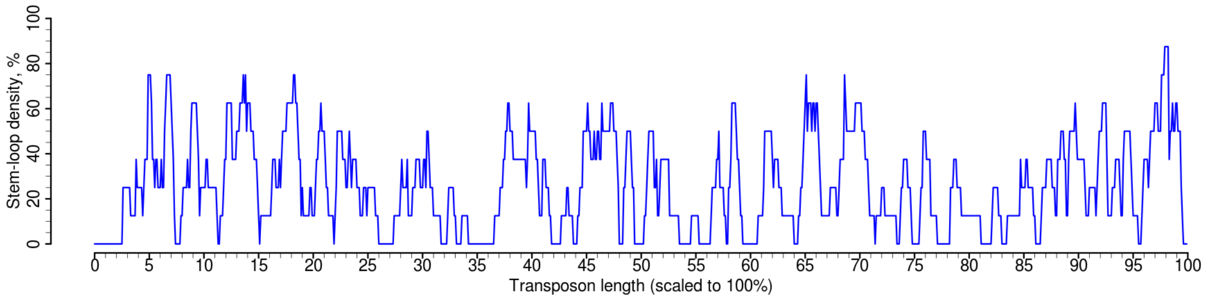

Supplement: Additional file 3: — Stem-loop coverage profiles for 27 L1 subfamilies from (Khan, Smit et al. [53]) (PDF 2803 kb) [file 12864_2016_3344_MOESM3_ESM.pdf]
