# Supplementary figures and images for: Conserved 3′ UTR stem-loop structure in L1 and Alu transposons in human genome: possible role in retrotransposition
Source: BMC Genomics. 2016 Dec 3;17:992. doi: 10.1186/s12864-016-3344-4 (PMC5135761; doi:10.1186/s12864-016-3344-4)

5'UTR

ORF1

ORF2

3'UTR

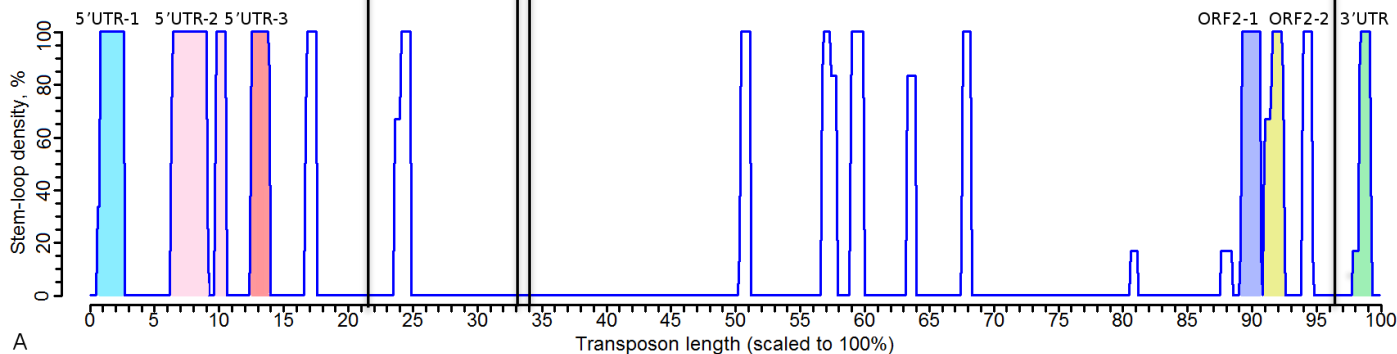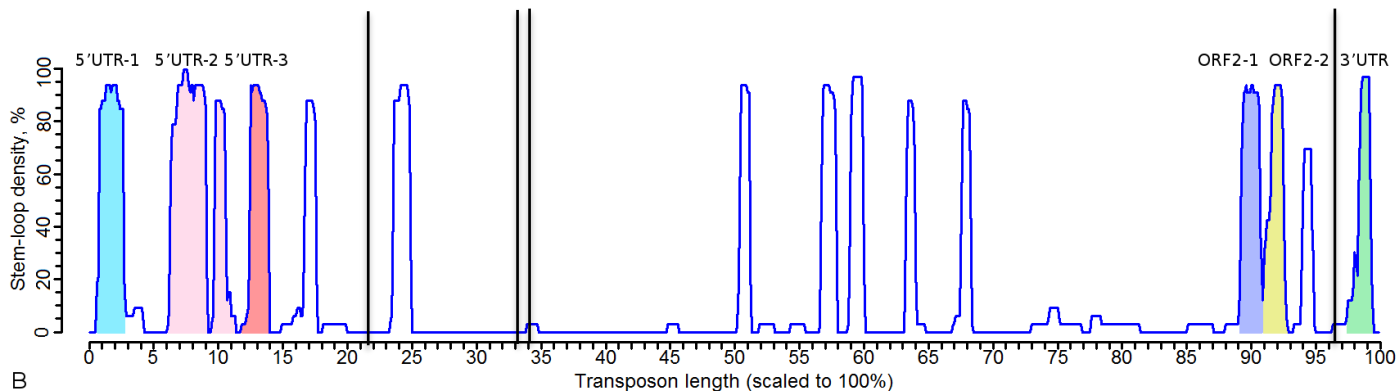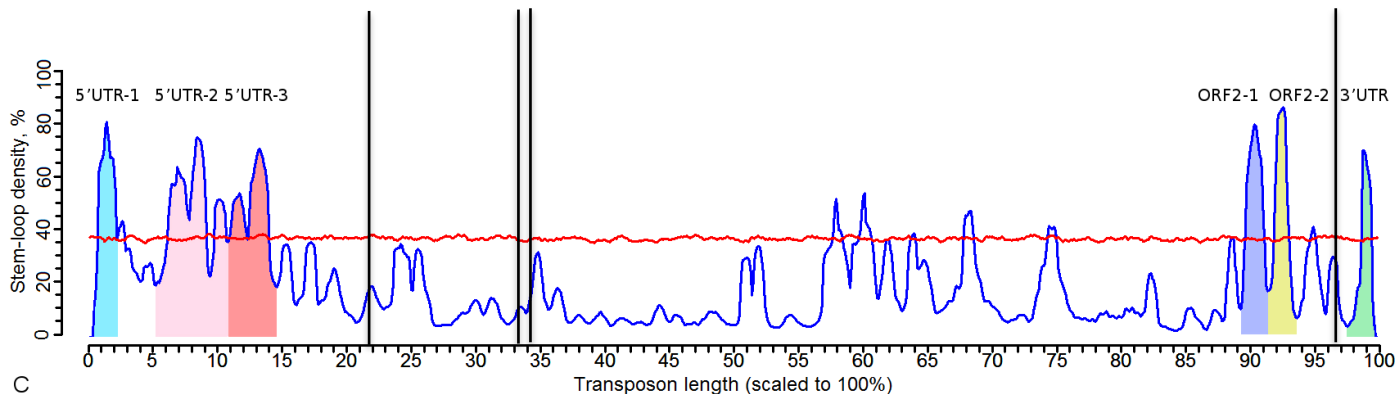

Supplement: Additional file 4: — Stem-loop profiles for active and highly conserved L1 transposons: (A) set of 6 hottest L1 transposons reported as active in (Brouha, Schustak et al. [3]); (B) set of 33 active L1 transposons reported as active in (Brouha, Schustak et al. [3]); (C) set of 6622 highly conserved L1 transposons (see Methods for selection criteria). (PDF 221 kb) [file 12864_2016_3344_MOESM4_ESM.pdf]

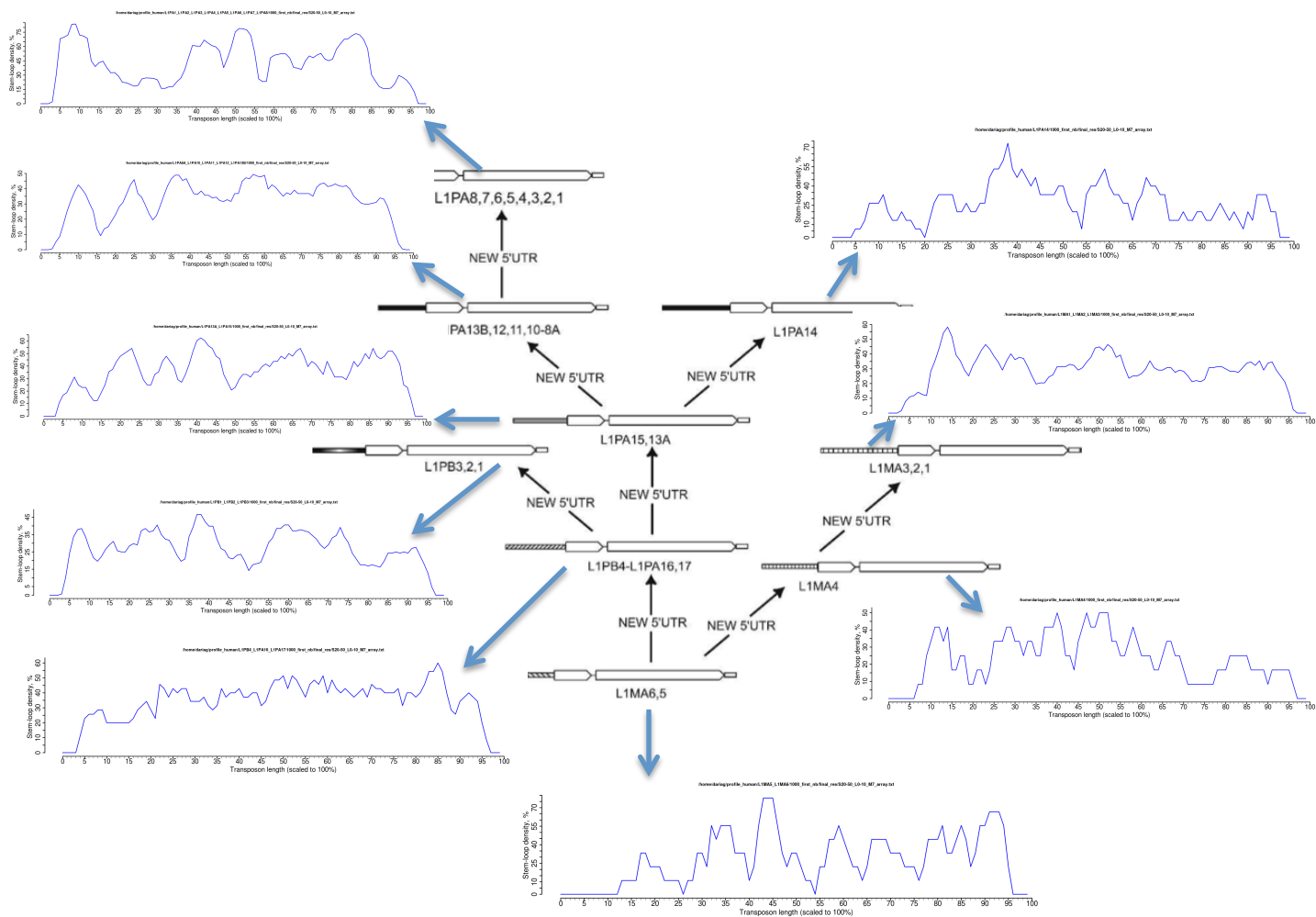

Supplement: Additional file 5: — Stem-loop profiles for 5′UTR regions of groups of L1 subfamilies having one type of 5′UTR as it was proposed in (Khan, Smit et al. [53]). (PDF 703 kb) [file 12864_2016_3344_MOESM5_ESM.pdf]

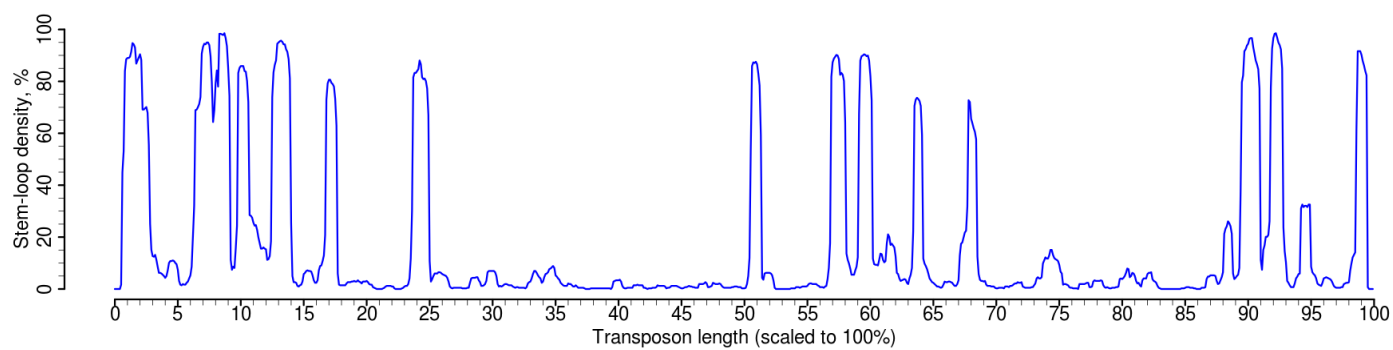

A

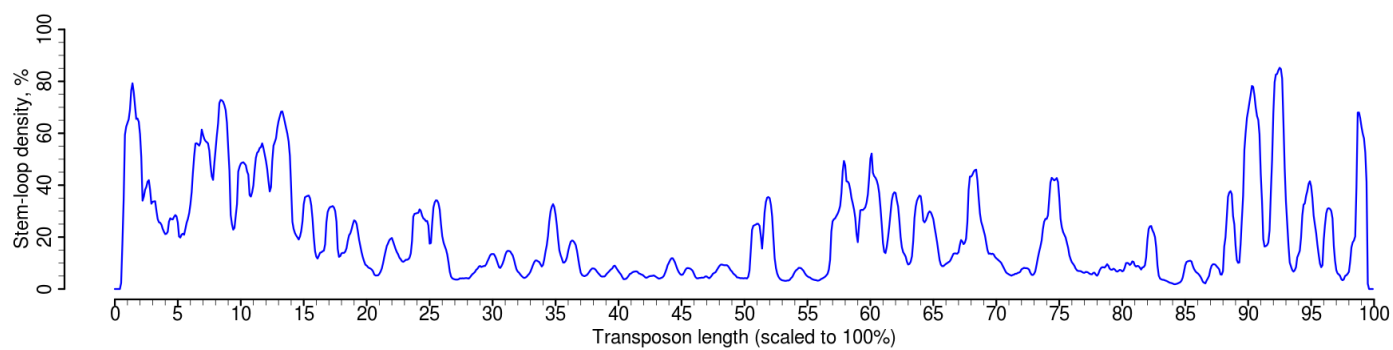

B

Supplement: Additional file 7: — Stem-loop profiles for intact or non-intact ORFs: (A) set of L1 transposons with intact ORFs; (B) set of L1 transposons with frameshift mutations in ORFs. (PDF 217 kb) [file 12864_2016_3344_MOESM7_ESM.pdf]

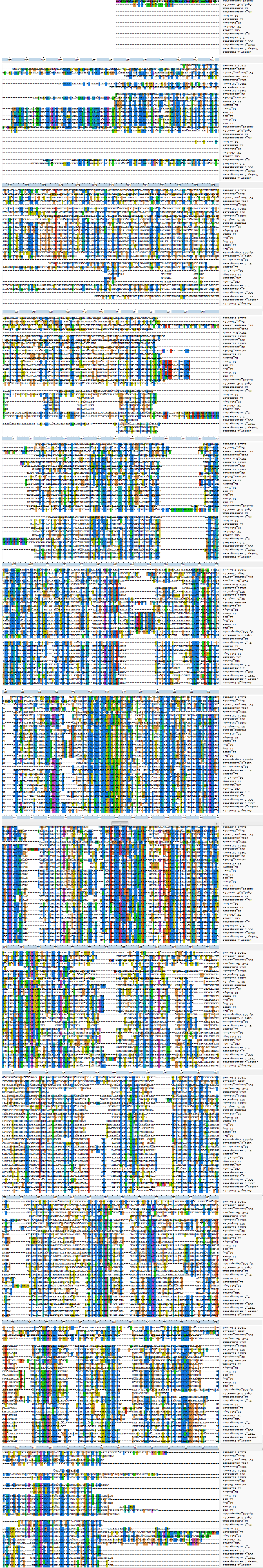

Supplement: Additional file 9: — Full alignment of ORF2 protein taken from different types of LINE, it is not limited to L1 and includes L2, R1, R2, CR1, I, Jockey, and others. (PNG 1346 kb) [file 12864_2016_3344_MOESM9_ESM.png]
